# Metagenomic analysis of the biofilm community at the oxic-anoxic interface of a deep-underground saline spring at the Baksan Neutrino Observatory

**DOI:** 10.1128/spectrum.02103-25

**Published:** 2026-02-27

**Authors:** Kirill Tarasov, Mikhail Zarubin, Alena Yakhnenko, Albert Gangapshev, Elena Kravchenko

**Affiliations:** 1 Dzhelepov Laboratory of Nuclear Problems, Joint Institute for Nuclear Research 68634, Dubna, Russia; 2 Institute for Nuclear Research, Russian Academy of Sciences 54744, Moscow, Russia; Universitat Wien, Vienna, Austria

**Keywords:** microbial community, deep-underground, metagenome

## Abstract

In this work, the first-ever metagenomic study of the microbial community from the deep-underground saline spring located at the Baksan Neutrino Observatory (BNO) (Kabardino-Balkaria, Russia) is presented. Using the metagenomic approach, we obtained 19 metagenome-assembled genomes (MAGs) attributed to the phyla *Pseudomonadota* (the dominant phyla), *Planctomycetota*, *Myxococcota*, *Nitrospirota*, *Gemmatimonadota*, *Armatimonadota*, and *Cyanobacteriota*. Archaea are generally absent in the metagenome. The microbial community of the Baksan Neutrino Observatory demonstrates a high metabolic diversity, including carbon dioxide-fixing, methane-oxidizing, dinitrogen-fixing, nitrate- and iron-reducing, anammox, nitrifying, and predatory bacteria. Hydrogen, methane, ammonia, and reduced iron compounds, present in the ecosystem, provide energy for primary organic production. The abundance and diversity of bacteria capable of carrying out various stages of the nitrogen cycle suggest that nitrogen compounds are of great significance for microbial community metabolism. On the basis of the Genome Taxonomy Database Toolkit classification of MAGs and comparison to the closest RefSeq genomes, we have identified six new genera, with the proposed names—“*Candidatus* Jinrbaksania,” “*Candidatus* Neutrinellum,” “*Candidatus* Jinrextremum,” “*Candidatus* Inrsubterrania,” “*Candidatus* Inralta,” and “*Candidatus* Neutrinobacter.” Comparative analysis with metagenomes of microbial communities from the deep underground granitic sites and karst caves reveals that the BNO microbial community represents a unique transitional ecosystem on the boundary between the deep anoxic and surface aerobic biosphere.

**IMPORTANCE:**

The deep biosphere makes up 12–20% of the Earth’s biomass and is poorly studied due to its inaccessibility. To date, only a few metagenomic studies of local deep biospheres have been performed in Russia. The Baksan Neutrino Observatory (BNO) is a deep-underground laboratory, with some abandoned tunnels. One of them hosts a mineral spring saturated with volcanic gases from the peripheral magma chamber of Mount Elbrus. The metagenomic analysis of the biofilm from this mineral spring has revealed the presence of unique microbial community whose composition occupies a transitional position between deep-underground microbial communities and communities of karst caves. We believe that this study of the microbial metagenome of the saline spring of the BNO will make a valuable contribution to understanding the composition and functioning of microbial communities formed at the oxic-anoxic interface.

## INTRODUCTION

The deep biosphere is one of the least studied ecosystems, despite the fact that, according to the most recent estimates, it accounts for 12–20% of the Earth’s biomass (~10^30^ cells) (1, 2). It is dominated by uncultivated organisms (>99.9%) and thus is entitled “dark matter of the biological universe” (3, 4). The predicted depth limit for life approaches 5–6 km underground, with organisms currently known dwelling at a depth of 4.5 km (5–7). The critical limiting factors for organisms at such depths are the temperature of up to ~120°C and decrease in permeability and porosity of rocks necessary to provide the flow of nutrients (1, 8). There is not much data about microbial communities from the depths greater than 2 km due to access difficulties, and thus every new research opportunity provides a valuable contribution to overall knowledge about the structure of deep-underground microbial communities.

Most of the data about deep-underground ecosystems were obtained from mines, drilling sites, or various underground facilities (2). Among the most extensively studied sites are gold mines of the Witwatersrand Basin in South Africa, with samples taken from the depths of up to 3.5 km (9, 10), and tunnels and boreholes of the Fennoscandian Shield with depths of up to 450 m studied at the Äspö Hard Rock Laboratory, Sweden (11, 12). Also, several studies were carried out in granite-rock environments of Outokumpu, Finland (13–15); in drill holes intended for future deployment of geothermal power plants in Otaniemi, Finland (7); in the future nuclear waste storage of the Grimsel Test Site, Switzerland (16); at the Mizunami Underground Research Laboratory, Japan (17, 18); in rocks of Deccan Traps, India (19, 20); and in nuclear waste disposals in Siberia, Russia (21, 22).

Deep-underground ecosystems are mainly inhabited by representatives of the phyla *Pseudomonadota* (mostly *Gamma-* and *Betaproteobacteria*), *Bacillota* (mostly *Clostridia*), *Bacteroidota*, and *Chloroflexota* (2), and less often *Acidobacteriota*, *Actinobacteriota*, *Desulfobacterota*, *Thermotogota*, *Nitrospirota*, *Patescibacterota*, *Armatimonadota*, *Elusimicrobiota*, *Spirochaetota*, *Myxococcota*, *Campilobacterota*, and *Methylomirabilota*, though the abundance of a particular phylum can vary greatly due to the geologic composition of the host rock (8). These phyla are often represented by extremophilic members able to survive high temperature, high pressure, low concentrations of carbon compounds, high concentrations of salts and heavy metals, and low or high pH levels (23), and thus are of particular interest for biotechnology, for example, for ore bioleaching or bioremediation.

Although most studies have focused on anoxic conditions in boreholes, microbial life at the oxic interface represents a critical and understudied ecological transition zone. These oxic-anoxic interfaces, often marked by biofilm formation, host specialized microbial communities that mediate key biochemical processes and serve as the first responders that catalyze the oxidation of deep-derived hydrogen, methane, sulfide, and ferrous iron (24, 25).

In Russia, there are only a few deep-underground sites (namely, oil wells 2.8 km deep, radioactive waste sites ~0.7 km deep, and Caucasian mineral water basin wells 0.2–1 km deep), where microbiome studies have been conducted (21, 22, 26–28). In this work, we present the metagenomic analysis of the microbial community of a hydrothermal spring formed at the contact of deep underground anoxic waters with air at a depth of about 2 km in the farthermost part of the tunnel of the Baksan Neutrino Observatory of the Institute of Nuclear Research of the Russian Academy of Sciences (BNO of INR of RAS) in the Neutrino village, Kabardino-Balkaria, Russia. It is an underground facility located in the Baksan Gorge, approximately 22 km to the east of Mount Elbrus. Due to the proximity of the dormant stratovolcano Elbrus, and particularly to its peripheral magma chamber, the ecosystem of the BNO spring is significantly impacted by high temperatures and emission of volcanic gases (29–31). On the basis of metagenomic sequencing, we have assessed the composition of the microbial community inhabiting the hydrothermal spring, evaluated the metabolism of its members, and proposed the model of energy metabolism in this ecosystem, including the potential of using inorganic compounds of deep origin as a primary energy source for the microbial community. Furthermore, we contextualize our findings by comparing this biofilm microbial community with microbial communities from other deep granitic environments and microbial communities inhabiting cave ecological niches.

We hope that our study, along with other metagenomic studies selected for this analysis, will provide information for future meta-analyses that will describe a global panorama of microbial diversity in deep-subsurface microbial communities, their ecological dynamics, evolution, and the role in nutrient cycling.

## MATERIALS AND METHODS

### Sampling

The biofilm samples were taken in an unused part of the farthest BNO tunnel from a mineral spring (41°C) at a distance of ~4.2 km from the entrance (43°16′32.5″ N 42°41′30.3″ E) and ~2 km below the surface between the summits Andyrchi (3,937 m.a.s.l.) and Kurmychi (4,045 m.a.s.l.) (Fig. S1). The samples were collected at the foot of mineralized spring outlet into sterile tubes, frozen at −20°C, and transported to the laboratory, where they were stored at −80°C.

### Physical and chemical analysis of water

Physical and chemical analysis of water was carried out in the Analytical Center of the Faculty of Chemistry of Moscow State University. Hardness and elemental concentrations were assessed by ICP-AES; pH was measured by potentiometric titration; conductivity was measured with a conductometer HI 98311; mineralization was assessed by the gravimetric method; concentrations of inorganic ions and compounds were measured by colorimetric titration, spectrophotometry, and ion chromatography.

### DNA isolation and sequencing, assembly, binning, and annotation, and derivation of 16S/18S genes

This part of the study is described in all details in Tarasov et al. (32). Briefly, the DNA was extracted using the GeneJET Genomic DNA Purification Kit (Thermo Scientific). Libraries for sequencing were prepared using the Hyper Library, Kapa Biosystems (Roche). Sequencing was performed on the Illumina Miseq platform with the TruSeq kit. The reads were processed with bcl2fastq and trimmed using HTStream (https://github.com/s4hts/HTStream). The read quality was assessed using FastQC and MultiQC (33, 34). Contigs were assembled with MetaSPAdes (35). Binning was conducted using MetaBAT2, and the bin quality was assessed using CheckM (36, 37). The bin classification was performed with the Genome Taxonomy Database Toolkit (GTDB-Tk) (38). The 16S/18S rRNA genes were reconstructed from raw reads and classified using the RiboTaxa pipeline (39).

The percentage of different groups was calculated as follows. Since the microbes with longer genomes get more aligned reads to their genome, we adjusted the values of the mapped reads accordingly. For each metagenome-assembled genome (MAG), we have normalized the quantity of the mapped reads by the length of that MAG. The abundance percentage of different groups of organisms was calculated as follows:

1. All the reads were mapped to the obtained MAGs using the BWA mapper.
2. The mapped reads with the quality greater than 60 (*P ≤* 0.000001) were counted using SAMTOOLS.
3. The completeness and contamination values derived with CheckM for each MAG were used to estimate the true length of the genome according to the formula:

${\text{true length}}_{i}={\text{MAG length}}_{i}*\frac{100}{\left(1+\frac{{\text{contamination}}_{i}}{100}\right)*{\text{completeness}}_{i}}$

- Then the values of the mapped reads were normalized by the truelengths of the genomes:

$${\text{mapped reads normalized}}_{i}=\frac{{\text{mapped reads}}_{i}}{\text{ true }{\text{length}}_{i}}$$

The percentage of the *i*-th MAG was calculated as:


$${\text{ percentage}}_{i}=\frac{{\text{ mapped reads normalized}}_{i}}{\sum_{j=1}^{N}{\text{ mapped reads normalized}}_{j}}$$

**Fig 1:**
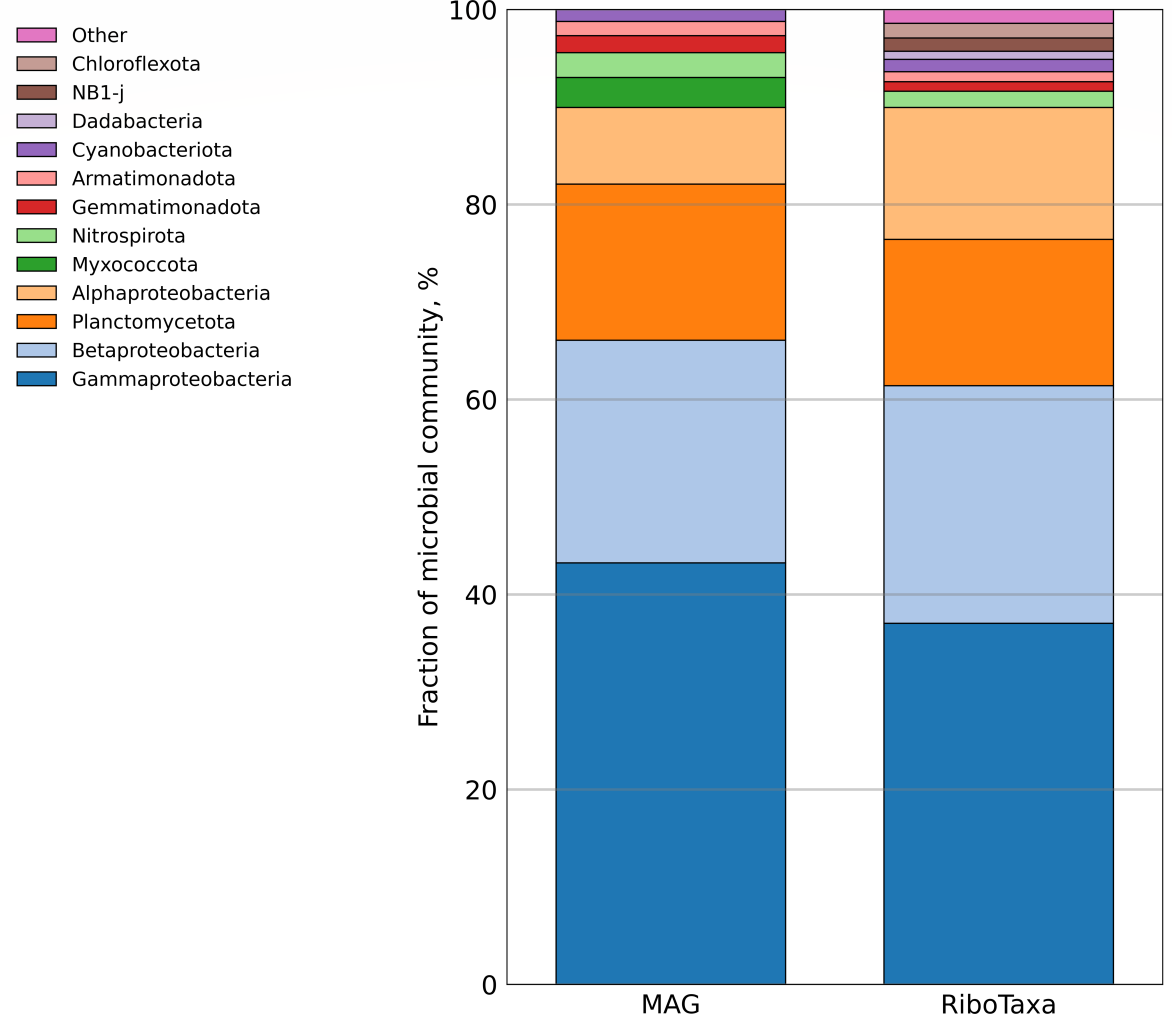
Relative abundance of various groups of microorganisms in the metagenome (MAG—based on 19 selected MAGs, RiboTaxa—based on reconstruction of SSU rRNA gene sequences from the raw reads with the abundances of phyla > 0.5%). Classes of the *Pseudomonadota* phylum are represented separately as *Alphaproteobacteria*, *Betaproteobacteria*, and* Gammaproteobacteria*.

RiboTaxa percentages were left as produced by the tool. The produced taxonomy was adjusted according to the LPSN DSMZ database (updated with ICNP). The cutoff for phylum presence was selected at 0.5%. Lineages with the abundances below this cutoff are represented in Fig. 1 as “Other.”

### Determination of metabolic pathways

Screening for functional traits, enzymes, and gene clusters for the synthesis of secondary metabolites was performed using the tools: METABOLIC v4.0 (40), dbCAN3 v4.0.0 (41), FeGenie v1.2 (42), and antiSMASH v7.1.0 (43).

### Phylogenetic analysis

For each MAG that was putatively designated as a new genus in GTDB-Tk, it was compared with the closest RefSeq assemblies. On the trees produced by GTDB-Tk, closest relatives that came from the RefSeq database were located, then average nucleotide identity (ANI) values were calculated using Pyani software (44), resulting ANI values were added on the tree. The trees were drawn using the ete3 library (45).

### Comparison of deep metagenomes of granitic rocks and caves

For each deep granitic or karst cave site (described in the Tables S1 and S2) the abundances of phyla were compiled as a single table. The map of the sites is given in Fig. S2. Metagenomes from these sites were compared based on Bray-Curtis dissimilarities. Microorganism abundances were represented at the phyla level, except for the *Pseudomonadota* phylum, which was represented at the class level (*Gammaproteobacteria*, *Alphaproteobacteria*, and *Betaproteobacteria*), and *Archaea*, which were represented at the domain level. The formula for Bray-Curtis dissimilarity is given below:

$Bray-Curtis\;dissimilarity\;between\;sites\;A\;and\;B=\;\frac{\sum_{i=1}^{N}\left|p_{iA}-p_{iB}\right|}{\sum_{i=1}^{N}\left|p_{iA}+p_{iB}\right|}$, where *p_iA_* denotes the percentage of the *i*-th microorganism phylum on site A.

## RESULTS

### Sampling site and water chemistry

Previous monitoring of the gas composition in the far end of the unused BNO tunnel revealed that releases of gases are in agreement with seismic activity of the region and carbon dioxide is enriched with ^13^C (29–31, 46). The more recent study confirmed the deviations in the isotope composition of the BNO spring water from the Global Meteoric Water Line, as well as the overall enrichment of gases with heavy isotopes (^13^C, ^18^O, ^17^O, ^3^Не, ^4^Не, and ^2^H), which led the researchers to the conclusion that gases in the BNO spring are of magmatic genesis (47).

The water from the BNO spring was moderately mineralized (8.1 g/L), with the pH value of 7.3 (Table 1). The most abundant dissolved elements were Cl, Na, K, B, Mg, Li, Ba, and Fe. The boron concentration was 180 mg/L, one of the highest values among sites where microbial diversity studies were conducted (48, 49).

**TABLE 1:** Physical and chemical characteristics of the water of the BNO spring

Parameter	Value
pH	7.30
Total hardness, mg/L	1,050
Conductivity, mS/cm	11.57
*T*, ^o^C	41
Fe, mg/L	1.30
Mn, mg/L	0.02
Сu, mg/L	<0.001
Pb, mg/L	<0.001
Ag, mg/L	<0.005
Ba, mg/L	4.00
Be, mg/L	<0.0001
W, mg/L	<0.01
Co, mg/L	<0.001
Cr, mg/L	<0.001
Mo, mg/L	<0.001
Ni, mg/L	<0.001
Sr, mg/L	2.70
V, mg/L	<0.001
Zn, mg/L	<0.005
B, mg/L	180
As, mg/L	<0.005
Cd, mg/L	<0.0001
Li, mg/L	7.40
Na, mg/L	2,030
K, mg/L	220
Ca, mg/L	170
Mg, mg/L	160
Al, mg/L	<0.01
Si, mg/L	46
Hg, mg/L	<0.01
HCO_3_, mg/L	2,870
CO_3_, mg/L	<6
NH_4_, mg/L	0.04
NO_2_, mg/L	0.04
F, mg/L	<0.3
Cl, mg/L	2,425
NO_3_, mg/L	<0.5
SO_4_, mg/L	<0.5
PO_4_, mg/L	<0.5
H_2_S, mg/L	<0.002
S, mg/L	<0.002
HS, mg/L	<0.002
Mineralization, mg/L	8,116

### Taxonomic identification and abundance of MAGs

A total of 42 MAGs of varying quality were recovered from the 4.66 Gbp of metagenomic sequences (32), and 19 MAGs were selected for future analysis on the basis of their CheckM quality assessments (Table 2). The criteria used for delineation of these MAGs were the following: the quality grade “high” or “medium” according to the classification by Bowers et al. (50), and the completeness of ≥90%. In total, these MAGs represented 81.53% of the entire-community metagenome, and the discussion below concerns only these 19 selected MAGs, unless otherwise explicitly stated.

**TABLE 2:** Main properties of 19 selected MAGs^*a*^

MAG	GTDB taxonomy	MAG size (Mb)	GC, %	Number of genes	Percentage	Completeness	Contamination
1	p__Proteobacteria;...;g__Nitrosomonas;s__	2.96	48.2	3,021	21.35	97.13	0.62
5	p__Planctomycetota;...;f__Brocadiaceae;**g__**;s__	3.84	39.8	4,019	1.01	98.9	2.75
6	p__Proteobacteria;...;g__Limibaculum;s__	3.87	68.7	3,817	5.48	99.5	0
14	p__Cyanobacteria;...;f__Vampirovibrionaceae;**g__**;s__	3.57	58.2	3,455	1.24	96.58	1.28
15	p__Proteobacteria;...;s__Methylocaldum_sp002005105	4.96	58.2	5,412	1.68	96.04	4.37
16	p__Nitrospirota;...;f__UBA8639;**g__**;s__	3.73	52.9	4,498	0.87	96.74	3.18
17	p__Planctomycetota;...;g__UBA5793;s__	4.26	66.9	3,804	3.45	94.32	1.14
18	p__Myxococcota_A;...;g__CAADGG01;s__	4.69	70.1	4,455	1.86	95.91	1.94
19	p__Proteobacteria;...;f__Wenzhouxiangellaceae;**g__**;s__	2.7	65	2,502	40.89	97.1	0.36
20	p__Armatimonadota;...;s__J051_sp003695835	2.58	61.1	2,481	1.45	93.21	2.78
22	p__Planctomycetota;...;g__J020;s__	2.88	66.6	2,684	7.19	98.86	0
24	p__Proteobacteria;...;s__Tatlockia_maceachernii	3.31	40.6	3,305	0.66	94.74	3.7
25	p__Nitrospirota;...;f__Nitrospiraceae;**g__**;s__	4.42	62.5	4,809	1.68	96.76	5.45
29	p__Myxococcota;...;f__SG8-38;**g__**;s__	7.85	72.1	7,619	1.22	97.17	7.9
30	p__Planctomycetota;...;g__CAADGN01;s__	3.16	69.7	2,949	2.6	100	3.51
37	p__Proteobacteria;...;s__PRO5_sp003577275	3.39	61	3,241	2.38	99.5	0
40	p__Planctomycetota;...;g__Kuenenia;s__	3.74	41	4,092	1.76	97.8	1.65
41	p__Proteobacteria;...;g__Nitrosomonas;s__	2.99	49.1	3,231	1.48	92.51	2.32
42	p__Gemmatimonadota;...;g__SZUA-320;s__	4.16	66.8	4,163	1.74	97.8	2.2

^*a*^ The MAGs assigned to new genera are highlighted in bold.

According to the phylogenetic identification based on the GTDB classification of MAGs, which had a good correlation with the RiboTaxa 16S rRNA gene profiling (Bray-Curtis dissimilarity value of 0.11), the most abundant phylum was *Pseudomonadota* (73.92%), with *Gammaproteobacteria* comprising 44.71%, *Betaproteobacteria* 21.35%, and *Alphaproteobacteria* 7.86%. The second most abundant phylum was *Planctomycetota*, making up 16.02% of the microbial community. The other phyla were *Myxococcota* (3.08%), *Nitrospirota* (2.55%), *Gemmatimonadota* (1.74%), *Armatimonadota* (1.45%), and *Cyanobacteria* (1.24%) (Fig. 1). The phyla *Desulfobacterota*, *Chloroflexota*, and *Acidobacterota* were also represented in the metagenome as low-quality MAGs with the abundances of <0.1% and are not discussed further.

In the BNO spring, archaea representatives are almost absent. The traces of the ammonium-oxidizing archaea (phylum *Crenarchaeota*) are found only in the reconstructed SSU sequences and amount to 0.13% of the microbial community, while in the metagenome, including poor-quality MAGs, archaea are not represented.

#### Gammaproteobacteria

The most abundant phylum in the BNO microbial community is represented by *Gammaproteobacteria*, with MAG19 (40.89%), MAG15 (1.68%), and MAG24 (0.66%), accounting for 44.71% of the metagenome. MAG19 belongs to the family *Wenzhouxiangellaceae*, MAG15 to the genus *Methylocaldum,* and MAG24 to the species *Tatlockia maceachernii*.

The 16S rRNA gene of MAG19 is found to be 92% identical to the *Wenzhouxiangella marina* 16S rRNA gene. *Wenzhouxiangellaceae* are a family of obligatory aerobic, chemoheterotrophic, generally non-motile bacteria (51). It is interesting that all known *Wenzhouxiangellaceae* isolates are moderately halophilic neutrophiles found in the water of seas and hypersaline lakes and are frequently able to hydrolyze proteins and polysaccharides of living or dead cells of gram-positive bacteria in order to use them as a growth substrate (52). Genes involved in the process of oxidative phosphorylation are present, suggesting that the organism is aerobic. It is noteworthy that the genes of two types of cytochrome oxidase (*coxAB* and *ccoNOP*) are detected. MAG19 contains the gene of Ni-Fe hydrogenase of group 1, which indicates that the organism is able to use hydrogen as a source of energy. The iron reduction genes of the Mtr respiratory pathway and the huge number of genes of cytochromes used for reducing insoluble iron compounds are determined. This implies that in anoxic conditions, the organism could be able to perform oxidative phosphorylation with oxidized iron as an electron acceptor.

According to the results of the taxonomic analysis with GTDB-Tk and ANI calculations, we can classify MAG19 as a potential new genus, with the proposed name “*Candidatus* Jinrbaksania” (Table 2; Fig. S4).

MAG15 is taxonomically assigned to the genus *Methylocaldum*. The genus *Methylocaldum* includes the thermotolerant and thermophilic type-I methanotrophic bacteria which typically use methane or methanol as a sole source of carbon and energy (53, 54). In MAG15, the genes coding the key enzymes for methylotrophic carbon assimilation via the Ribulose-monophosphate pathway are found. MAG15 also contains the gene coding RubisCo, which indicates that it could fix carbon dioxide via the CBB cycle. The genes of Ni-Fe hydrogenases of groups 1 and 2 are found in the genome of MAG15, which signals that the organism is able to use hydrogen as a source of energy. Moreover, MAG15 contains the genes of the Mo-Fe nitrogenase complex, implying that the organism is able to fix dinitrogen.

MAG24 is identified as *Tatlockia maceachernii*, a common inhabitant of water environments (55). It is an aerobic bacterium that can withstand temperatures up to 60°C (56). The presence of various carbohydrate hydrolysis genes in MAG24 suggests that the bacterium is able to degrade cellulose, starch, and chitin. The genes coding oxidative phosphorylation enzymes and the key enzyme of the Entner—Doudoroff pathway are also present in MAG24.

#### Betaproteobacteria

*Betaproteobacteria* are represented with MAG1 (21.35%) and MAG41 (1.48%), both of which are classified down to the genus *Nitrosomonas,* obligatory chemolithoautotrophs responsible for oxidation of ammonia to nitrite, the first stage of the nitrification process. In addition to a number of genes required for nitrification, both MAGs contain urease genes indicating that urea could be used as an alternative source of ammonia.

MAG1 has the *amoC* gene of ammonium-monooxygenase. Although the *amoAB* genes encoding ammonium monooxygenase and the genes encoding hydroxylamine oxidoreductase are absent in the genome, they are present in the initial metagenomic data in two contigs that were not binned because of their short length (2,000 and 4,800 nucleotides). Since the genus is well established as ammonium-oxidizing bacteria, we assume that these contigs are of the *Nitrosomonas* origin. The nitrite and nitric oxide reductase genes are present. It is well known that the *Nitrosomonas* genus is able to fix carbon dioxide via the CBB cycle, and indeed, in MAG1, the genes encoding RubisCo form I are detected. It is curious that Ni-Fe hydrogenase of group 3, also contained in MAG1, is typical of hyperthermophilic methanogens (57) and can operate in either direction. The genes encoding oxidative phosphorylation enzymes are found. Two copies of the iron-oxidizing *Cyc1* protein are also found, suggesting that the organism can utilize reduced iron as an energy source.

The metabolic potential of MAG41 quite resembles that of MAG1. It also contains the RubisCo form-I gene of the CBB cycle, the *amoC* gene of ammonia-monooxygenase enzyme, and the nitrite and nitric oxide reductase genes. Like MAG1, MAG41 contains oxidative phosphorylation enzyme genes. Ni-Fe hydrogenase of group 3 and urease genes are present just like in MAG1. Same as MAG1, MAG41 contains two copies of the *Cyc1* gene also suggesting it could utilize reduced iron.

#### Alphaproteobacteria

*Alphaproteobacteria* are represented with two MAGs: MAG6 (5.48%), classified down to the genus *Limibaculum,* and MAG37 (2.38%), classified down to the order *Sphingomonadales*. There is also a poor-quality MAG classified as the *Methylocella* genus (type-II methanotroph), as well as the multiple 16S and 28S rRNA gene sequences of the *Rhodobacteraceae* and *Beijerinckiaceae* families in a highly chimeric MAG32. The RiboTaxa tool also produces several 16S and 23S rRNA gene sequences of *Beijerinckiaceae*. All this evidence suggests that there are also type-II methanotrophs in the microbial community, though they account for less than 0.6% of it.

The *Limibaculum* is an aerobic non-motile bacterium of the *Rhodobacteraceae* family, which comprises aquatic bacteria (58). *Rhodobacteraceae* are a striking family because of the ability for horizontal gene transfer via unique virus-like particles, gene transfer agents (GTAs). Indeed, the GTA operon is present in MAG6. The abundance of genes coding C1 compound conversion enzymes points out that C1 compounds play a significant role in the life cycle of this bacterium. It can detoxify formaldehyde with these enzymes or incorporate C1 compounds into its own organic compounds via the serine cycle (the genes of its key enzymes are found). The organism is able to utilize short-chain haloacids due to the presence of 2-haloacid dehalogenase genes. A number of genes of arsenic compound conversions indicate that MAG6 can possess the ability to respire arsenate.

*Sphingomonadales* are an order of mostly chemoorganotrophic bacteria with some photoheterotrophic members (59). They are known for their ability to decompose a diverse set of organic compounds and thus are applied in biotechnology (59).

MAG6 and MAG37 contain genes for oxidative phosphorylation. In addition, MAG37 comprises a number of genes for aromatic degradation and degradation of cellulose, chitin, and starch and for respiration using ferric iron as an electron acceptor.

#### Planctomycetota

*Planctomycetota* are represented with five MAGs: MAG22 (7.19%), MAG17 (3.45%), MAG30 (2.6%), MAG40 (1.76%), and MAG5 (1.01%). *Planctomycetota* account for 16.01% of the metagenome, being the second most represented phylum. Three MAGs are assigned to the *Phycisphaeraceae* family (MAG30 to the genus *Candidatus CAADGN01*, MAG22 to the genus *Candidatus J020*, and MAG17 to *Candidatus UBA5793*), and two MAGs are assigned to the *Brocadiaceae* family (MAG5 is classified down to the family level and MAG40 is classified down to the genus *Kuenenia*).

*Phycisphaeraceae* are a highly represented family of *Planctomycetota* accounting for 13.24% of the whole metagenome. These bacteria are typically aerobic heterotrophs, able to degrade various complex organic substrates. They also possess a higher GC content (~73%) compared to other *Planctomycetota* (60). MAG22 contains the genes necessary for utilization of starch (alpha-amylase, gluco-amylase, and isoamylase). The genes for utilization of amino acids, fatty acids, other polysaccharides (except starch), and aromatic compounds are not detected. The genes involved in the oxidative phosphorylation chain are present. The gene of 2-haloacid dehalogenase is present, suggesting that the organism is able to utilize short-chained haloacids (two to four atoms). There is no evidence of the presence of genes coding enzymes for the oxidation of sulfur and nitrogen compounds, as well as of dissimilatory reduction enzymes, which indicates that the organism is most likely an aerobic chemoorganotroph.

MAG30 and MAG17, unlike MAG22, contain a few genes for the degradation of aromatic compounds. In addition, MAG17 has genes for utilization of starch, chitin, and l-lactate. MAG30 and MAG17 possess the dissimilatory nitrate reduction genes, suggesting that these two organisms could use nitrate as a terminal acceptor of electrons during respiration. Moreover, all three MAGs comprise a number of the different nitrogen compound reduction genes; therefore, it is probable that the *Phycisphaeracea* members of the microbial community play a significant role in the reduction of such compounds. Both MAG17 and MAG30 (just as MAG22) have the genes encoding enzymes of the oxidative phosphorylation chain. In addition, MAG30 contains 2-haloacid dehalogenase, the same as MAG22.

All three MAGs comprise genes coding iron reduction enzymes, suggesting that they may be able to use iron as an electron acceptor. MAG17 contains a total of 24 iron reduction genes (*FmnB*-1, *Ndh2*-2, *DmkB*-1, *DFE_0448*-3, *DFE_0449*-3, *DFE_0451*-3, *DFE_0461*-5, *DFE_0462*-5, and *DFE_0463*-1) while MAG22 and MAG30 have three and four iron reduction genes, respectively (MAG22: *DmkB*-2, *DFE_0451*-1; MAG30: *DmkB*-2, *FmnB*-2). It is possible that the mentioned members of the microbial community are chemoorganotrophs, with the potential of using nitrates and oxidized iron compounds for respiration.

The family *Brocadiacea* contains the only known anaerobic ammonia-oxidizing bacteria. Typically, they use nitrites as electron acceptors, instead of oxygen, and produce molecular nitrogen (60). Both MAG5 and MAG40 have the genes coding the key Wood-Ljungdahl pathway enzymes. It is a typical pathway for carbon dioxide fixation in anammox bacteria. Hydrazine synthase genes are absent in the MAGs, though they are present in the metagenomic data in unbinned short contigs. In MAG40, the genes of hydrazine dehydrogenase and cytochrome C oxidase are detected. Though it is not common for anaerobic bacteria to possess cytochrome genes, there is a recent report of expression of this type of cytochrome in anammox bacteria to respire oxygen produced in syntrophic conditions by algae (61). The presence of iron reduction genes suggests that anammox members of the microbial community could conduct the feammox process using ferric iron as an electron acceptor. MAG5 also contains the gene of Ni-Fe hydrogenase of group 3 which is typical of hyperthermophilic methanogens (57) and can operate in either direction.

According to the results of the taxonomic analysis using GTDB-tk and ANI calculations, we can classify MAG5 as a potential new genus, with the proposed name “*Candidatus* Jinrextremum” (Table 2; Fig. S4).

#### Nitrospirota

The phylum *Nitrospirota* is represented by MAG25 (1.68%) and MAG16 (0.87%). MAG25 is classified down to the family *Nitrospiraceae*, and MAG16 down to the order *Nitrospirales*. In nature, *Nitrospirota* are predominantly nitrite oxidizers (60) responsible for the second stage of the nitrification process. The phylum contains the only known comammox bacteria, which are able to simultaneously conduct the first and second stages of nitrification (62). MAG25 and MAG16 comprise genes of subunits of nitrite oxidoreductase, though not of all its subunits. In addition, both MAGs have many genes involved in oxidative phosphorylation. The genes for oxidation of ammonium (*amo*) and hydroxylamine (*hao*) are not found in MAG25 and MAG16, indicating that the MAGs represent common nitrite oxidizers rather than comammox bacteria. Both MAGs contain genes coding the key enzyme of the reverse TCA cycle, the citrate lyase, indicating the ability to fix CO_2_. Sulfides and hydrogen can be used as electron donors in this process, and indeed, MAG25 (but not MAG16) comprises the sulfide-quinone oxidoreductase gene involved in oxidation of sulfides, as well as the genes encoding Ni-Fe hydrogenase.

According to the results of the taxonomic analysis using GTDB-tk and ANI calculations, we can classify MAG16 as a potential new genus, with the proposed name “*Candidatus* Inrsubterrania” (Table 2; Fig. S4).

According to the results of the taxonomic analysis using GTDB-tk and ANI calculations, we can classify MAG25 as a potential new genus, with the proposed name “*Candidatus* Inralta” (Table 2; Fig. S4).

#### Myxococcota

*Myxococcota* are represented with two MAGs: MAG18 (1.86%) classified down to the family *Polyangiaceae* and MAG29 (1.22%) classified down to the order *Polyangiales*. The phylum *Myxococcota* is regarded as having the largest genome among all bacteria, with a high GC content. Many representatives are able to form complex fruiting bodies. There are also a number of predatory bacteria among *Myxococcota* (63, 64).

MAG29 contains genes coding for subunits of aerobic carbon monoxide dehydrogenase. The reversible reaction catalyzed by this enzyme can provide the organism with carbon dioxide as a source of carbon for anabolic reactions. The organism is able to perform an almost complete pathway of reduction of nitrate to dinitrogen since almost all the necessary genes are present. The organism also contains genes for reduction of nitrite to ammonia and the gene of sulfur dioxygenase. In addition, the organism possesses genes involved in oxidative phosphorylation and is possibly capable of aerobic respiration. This organism is very likely able to respire using perchlorate as a terminal acceptor (the genes of perchlorate reductase and chlorite dismutase are found). In addition, a number of iron reduction genes are detected, which suggests that the organism is able to respire using oxidized iron.

According to the results of the taxonomic analysis using GTDB-tk and ANI calculations, we can classify MAG29 as a potential new genus, with the proposed name “*Candidatus* Neutrinellum” (Table 2; Fig. S4).

Genomic traits of MAG18 resemble those of MAG29 quite closely. However, MAG18, unlike MAG29, contains a number of genes involved in C1 metabolism, suggesting that the organism is able to utilize methanol and methylamine. Interestingly, MAG18 holds the nitric oxide reductase gene, missing in MAG29, which suggests that these two organisms can syntrophically reduce nitrate to dinitrogen. The sulfur dioxygenase gene is also present. In addition, MAG18 contains the Ni-Fe hydrogenase of the first type, which usually is O_2_-sensitive and operates with the preference for H_2_ oxidation (65). The presence of such an enzyme indicates that the organism can use dihydrogen (H_2_) as an alternative energy source. The organism is probably capable of aerobic respiration since oxidative phosphorylation genes are found. The present genes of iron oxidation and iron reduction suggest that the organism can use oxidized iron in respiration as an electron acceptor and reduced iron as a source of energy.

#### Armatimonadota

*Armatimonadota* are represented with a single MAG20 (1.45%), classified as a member of the order *Fimbriimonadales*. The organism is likely able to perform oxidative phosphorylation since a number of genes involved in the corresponding pathway are present. The genes of enzymes of the sulfur and nitrogen cycling are not detected; however, a number of iron reduction genes are found, suggesting that the organism is able to use iron for respiration.

#### Gemmatimonadota

*Gemmatimonadota* are represented with MAG42 (1.74%), and the closest bacterium (76.43% ANI) to it was isolated from marine hydrothermal sulfide sediments in the Atlantic Ocean (GenBank accession GCA_003246755.1). The organism contains genes for nitrate reduction and nitrous oxide reduction. The incomplete nitrate reduction pathway is quite typical of *Gemmatimonadota* (66). The genes of two types of Ni-Fe hydrogenases are found: those involved in the H_2_-evolution and those involved in the H_2_-uptake. It suggests that the organism can use hydrogen both as a reducing agent and as a stock for excess electrons. The genes of oxidative phosphorylation are partially represented. The presence of the genes coding for most parts of the electron transport chain suggests that the microorganism is able to perform oxidative phosphorylation, and the absent genes were not included at the binning stage. The organism can probably use arsenate for respiration since the gene of arsenate reductase is present. It also possesses a gene of arsenite methyltransferase, an enzyme involved in the production of trimethylarsine, in this case possibly for detoxification purposes. Iron oxidation genes of the Cyc2 pathway are present, as well as multiple iron reduction genes, suggesting that the organism participates in the iron cycling of the BNO spring.

#### Cyanobacteriota

*Cyanobacteriota* are represented with MAG14 (1.24%), classified down to the family *Vampirovibrionaceae*. It is a group of aerobic non-photosynthetic obligate epitopic algae parasites that contain the Type-IV secretion system (T4SS) used to “suck out” their prey. Indeed, MAG14 has a number of T4SS protein genes. It also comprises a lot of flagellum-associated protein genes*,* which assumes that MAG14 is motile. A number of aminotransferase genes, as well as the genes of cellulase, arabinosidase, alpha-amylase, isoamylase, and hexosaminidase, point out that MAG14 utilizes multicarbon compounds. MAG14 is capable of oxidative phosphorylation as it contains the genes of the TCA cycle and the electron transport chain. Since no sequences in the metagenome are classified as belonging to algae, we suggest that *Vampirivibrio* can prey on other bacteria in the microbial community just like the predatory bacterium *Bdellovibrio bacteriovorus* (67).

According to the results of the taxonomic analysis using GTDB-tk and ANI calculations, we can classify MAG14 as a potential new genus, with the proposed name “*Candidatus* Neutrinobacter” (Table 2; Fig. S4).

## DISCUSSION

### Biotechnological potential of the microbial community of the BNO deep-underground spring

**Fig 2:**
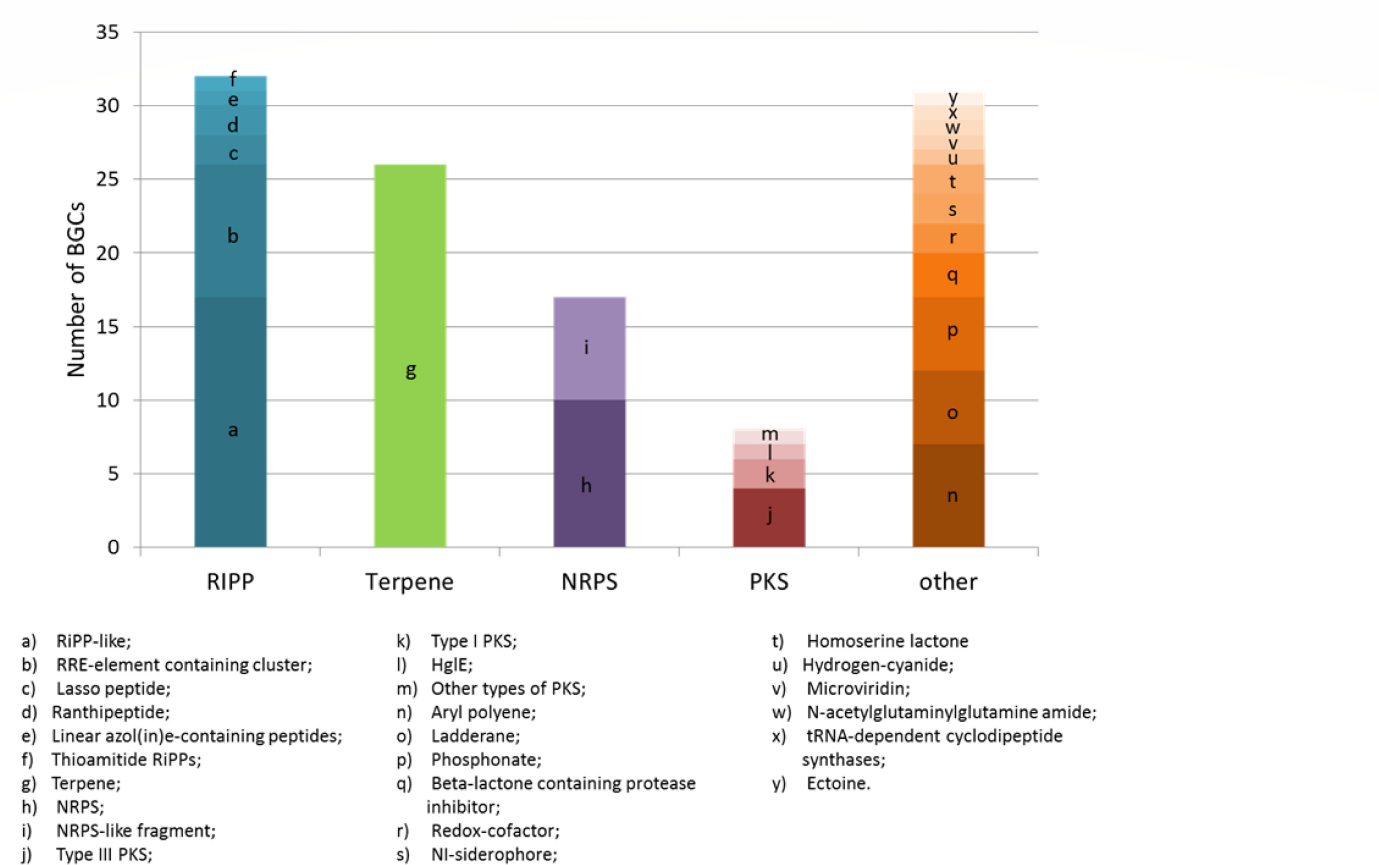
Secondary metabolite biosynthetic gene clusters found in the BNO metagenome data.

The search for new metabolic pathways in hard-to-reach natural microbial communities with the potential for applications in various fields, especially in medicine, biotechnology, and bioremediation, is highly relevant (68). A total of 114 biosynthetic gene clusters (BGCs) responsible for the synthesis of secondary metabolites are identified in the microbial community of the BNO spring. Out of 114 BGCs, 83 are of four major classes: 32 are the ribosomally synthesized and post-translationally modified peptides, 26 are terpene, 17 are nonribosomal peptide synthetases, and 8 are polyketide synthases. The remaining 31 clusters are labeled as “other,” including aryl polyene and ladderane (Fig. 2).

The KnownClusterBlast analysis reveals that among the identified BGCs, 17 are contained in the MIBiG 3 database: saccharide (MAG1), glycopeptidolipid NRP (MAG6), fosfomycin/Aminocoumarin (MAG14), hopene (MAG15 and MAG18), phenolic lipids (MAG15), vibrioferrin (MAG15), bisucaberin B (MAG16), aryl polyene (MAG18, MAG25, and MAG29), fengycin (MAG24), xiamycin (MAG25), carotenoid (MAG29), ectoine (MAG29), neoantimycin (MAG37), acyldepsipeptide 1 (MAG40), phosphinothricintripeptide (MAG42). The remaining 97 BGCs appear to be novel.

### Carbon metabolism

Three different pathways represent carbon dioxide fixation in the BNO spring: Calvin-Benson-Bassan cycle (MAG1 and MAG41*—Nitrosomonas*, MAG15*—Methylocaldum*), Wood-Ljungdahl pathway (MAG5 and MAG40*—Brocadiaceae*), and Reverse Tricarboxylic Acid cycle (MAG16 and MAG25*—Nitrospiraceae*). The organisms able to perform carbon dioxide fixation sum up to 29.83% of the microbial community, which is not unusual since carbonates are abundant in the BNO spring (2,870 mg/L) and carbon dioxide reaches 98.6% of the spring gas phase (47).

Methane-utilizing organisms are represented with type-I methanotrophs of the *Methylocaldum* genus (MAG15). Most likely, type II methanotrophs are also found in the BNO spring: there is a low-quality MAG which was classified down to the *Beijerinckiaceae* family, and *Methyloceanibacter* 16S rRNA of *Hyphomicrobiaceae* family is reconstructed from raw reads with the RiboTaxa tool. Altogether, these two MAGs (MAG15 and low-quality *Beijerinckiaceae* MAG) comprise up to 2.5% of the metagenome. We suppose that methanotrophic bacteria are one of the groups of primal producers of organic compounds in the BNO spring since methane of volcanic origin (47) is present in the spring gas phase in a substantial concentration (0.39%).

### Nitrogen metabolism

The following groups are present and contribute to the nitrogen cycle in the BNO spring—nitrogen-fixing bacteria, ammonium- and nitrite-oxidizing bacteria, anammox bacteria, nitrate reducers, bacteria able to degrade nitrogen-containing organic compounds.

The gene of nitrogenase is present in two MAGs: in MAG15 belonging to the type-I methanotroph of the *Methylocaldum* genus (1.68%) and in the low-quality MAG, which is classified down to the *Rhodocyclaceae* family. Taken together, these two MAGs comprise 2% of the microbial community. Nitrogen-fixing representatives are well known among type-I methanotrophs (69).

Nitrifying bacteria were detected, including ammonium-oxidizing (MAG1—21.35%, MAG41—1.48%), nitrite-oxidizing (MAG16—0.87%, MAG25—1.68%), and anammox bacteria (MAG5—1.01%, MAG40—1.76%). The abundance of ammonium-oxidizing organisms can result from significant amounts of the ammonium in the BNO spring (0.04 mg/L), and though the isotope analysis of that molecule in the BNO spring has never been conducted, the isotope analysis of other gases from the spring indicates their crustal and volcanic genesis (47).

Four MAGs capable of reducing nitrate are found: MAG17 (3.45%) and MAG30 (2.6%) of the *Phycisphaeraceae* family, MAG37 (2.38%) of the *Sphingomonadaceae* family, and MAG29 (1.22%) of the *Polyangiales* order. Also, the genes of nitrate reductases are identified in many low-quality MAGs.

The presence of nitrogen-fixing, anammox bacteria, and bacteria able to reduce nitrate suggests that zones with anoxic conditions persist in the biofilm community of the BNO spring.

### Sulfur metabolism

The genes for dissimilatory sulfate reduction are found only in three low-quality MAGs, which are not included in the final set. Taken together, these three MAGs comprise less than 0.2% of the BNO microbial community. It could be explained by predominantly aerobic conditions in the BNO spring, a low concentration of sulfates (<0.5 mg/L) compared, for example, to the Kidd Creek sites (31.7–59.5 mg/L) (70), as well as the preferences for nitrate and ferric ions as electron acceptors for anaerobic respiration.

Two MAGs, namely MAG15 (1.68%) of the *Methylocaldum* genus and MAG25 (1.68%) of the *Nitrospiraceae* family, are capable of utilizing both sulfides and elemental sulfur, and MAG6 (5.48%) of the *Limibaculum* genus is capable of utilizing only elemental sulfur as a source of energy. In addition, there are two groups of sulfur-oxidizing bacteria detected in the BNO community according to RiboTaxa classification*—Thiohalobacteraceae* and *Thiobacillaceae* families (Fig. 1) with the abundances of 0.5% and 1.1%, respectively. All this evidence suggests that there might be an ongoing process of reduced sulfur compound oxidation in the BNO spring.

### Iron metabolism

In total, 11 MAGs possess at least one of the following iron oxidation genes (*Cyc1*, *Cyc2*, *MtoA*, *FoxE*, *FoxZ*, and *MtrB*), including two of the most dominant MAGs, namely MAG19 (40.89%) of the *Wenzhouxiangellaceae* family and MAG1 (21.35%) of the *Nitrosomonas* genus. Altogether, those 11 MAGs (MAG1, MAG5, MAG6, MAG15, MAG18, MAG19, MAG24, MAG37, MAG40, MAG41, and MAG42) comprise 80.3% of the microbial community. Thus, it can be assumed that a continuous process of oxidation of divalent iron occurs in the BNO community; however, based on the data obtained, we cannot identify specific participants responsible for this process.

This suggestion is in accordance with a high concentration of iron in spring water (1.3 mg/L) (Table 1), as well as with the observation of brown insoluble sediments in proximity to the spring outlet, which are likely composed of ferric iron salts and hydroxides.

Some microbial community members are capable of iron reduction. The entire set of genes of the *MtrABC* pathway is identified in MAG19 (40.89%) of the *Wenzhouxiangellaceae* family, and two out of three genes, *MtrA* and *MtrB*, are identified in MAG18 (1.86%) of the *Myxococcota* phylum and in MAG37 (2.38%) of the *Sphingomonadaceae* family. The genes of different outer-membrane cytochromes, namely *DFE_448*, *DFE_449*, *DFE_450*, *DFE_451*, *DFE_461*, *DFE_462*, *DFE_463*, *DFE_464*, and *DFE_465* (according to reference 71), are found in MAG16, MAG17, MAG18, MAG19, MAG37, MAG5, MAG42, MAG20, MAG25, MAG16, and in the poorest-quality MAGs. These cytochromes provide the direct electron transport pathway for the reduction of insoluble iron compounds. And due to the ubiquity of such genes in MAGs, it is assumed that utilization of such compounds in respiration is widespread in anoxic zones of the spring.

**Fig 3:**
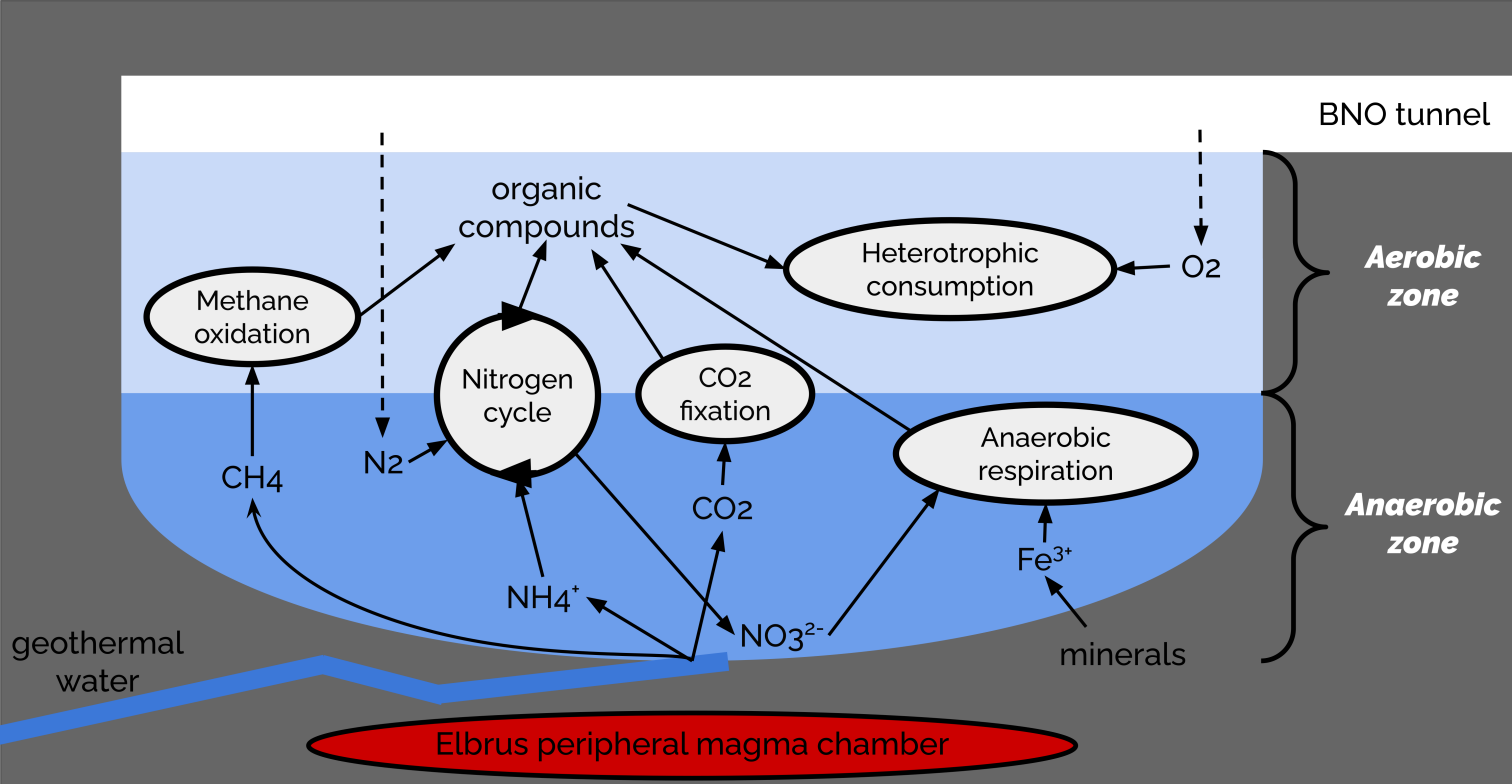
Model of energy metabolism in the microbial community of the BNO spring.

The resulting model for energy metabolism in the microbial community of the BNO spring is featured in Fig. 3.

To contextualize our results, we compare the microbial community of the BNO to the well-studied deep underground ecosystems (deep granitic underground sites and karst caves) using beta-diversity. Deep subsurface environments are characterized by darkness, oligotrophy, and stable conditions; however, deep granitic underground sites and karst caves may differ in oxygen regime (redox conditions) and exhibit variability in oxygen levels (anoxic, hypoxic, and oxic zones) (11, 72).

### Comparison of the BNO microbial community with microbial communities of deep underground granitic sites and karst caves based on beta-diversity (Bray-Curtis dissimilarity)

**Fig 4:**
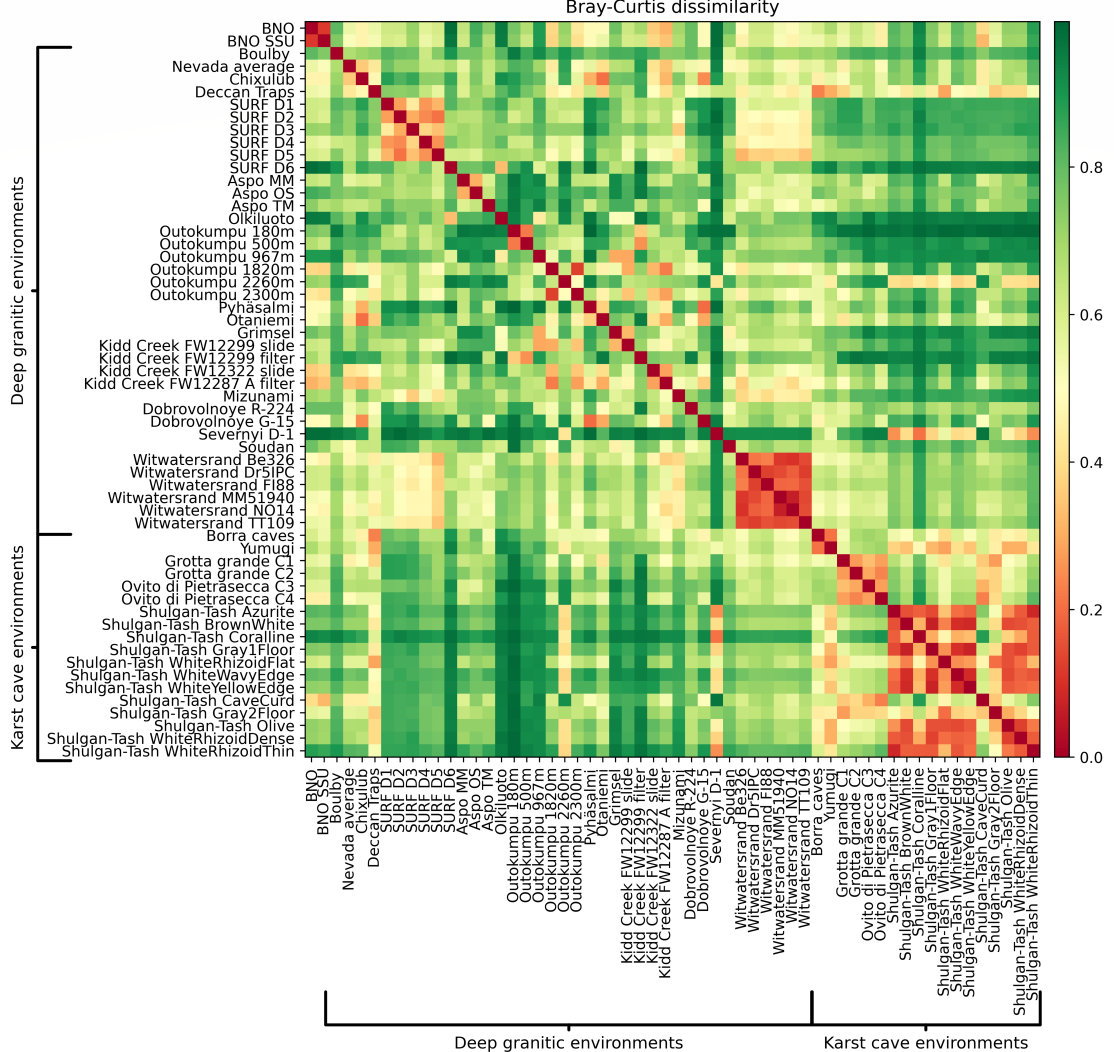
Bray-Curtis dissimilarities of the set of the selected deep granitic and karst cave microbiome studies. There are two entries for the BNO site: the “BNO” entry includes the abundances on the basis of the metagenomic data and the “BNO SSU” entry includes the abundances provided by RiboTaxa from the reconstructed 16S rRNA sequences.

We also calculated the Bray-Curtis dissimilarities based on the same data set (Table S2) (Fig. 4).

The microbial communities that are most similar to the BNO microbial community based on the Bray-Curtis dissimilarity coefficient are those from the Kidd Creek and Outokumpu mine data sets, and one from the Shulgan-Tash cave data set.

The five closest sites are Kidd Creek “FW12287 A filter” (*β* = 0.31), “FW12322 slide” (*β* = 0.36), Outokumpu 1820m (*β* = 0.4), Outokumpu 2300m (*β* = 0.43), and Shulgan-Tash “Cave Curd” (*β* = 0.4). The first four sites, like the BNO site, contain representatives of the *Alphaproteobacteria*, *Betaproteobacteria,* and *Gammaproteobacteria,* which make up 65–75% of the microbial communities, while the last one shares with the BNO site similar amounts of *Alphaproteobacteria*, *Gammaproteobacteria*, *Nitrospirota*, and *Planctomycetota* (a total of about 70%).

However, the Kidd Creek mine sites, unlike the BNO site, contain a vast portion of sulfate-reducing *Bacillota* representatives. This could be explained by anaerobic conditions of Kidd Creek waters, as well as by significantly higher levels of sulfates at these sites (30–60 mg/L compared to <0.5 mg/L at the BNO site) (70). Another distinctive trait of the Kidd Creek sites is the presence of members of the *Bacteroidota* phylum. There are 27.14% of them on the FW12322 site and 3.86% on the FW12287A site. Since the authors do not comment on metabolic features of *Bacteroidota*, which are classified down only to the phylum level, it is impossible to say why we observe them in the Kidd Creek microbial community and do not in the BNO microbial community.

Unlike the BNO site, the Outokumpu sites have substantial portions of members of the *Bacillota* phylum, namely 28% (1,820 m) and 22% (2,300 m). All classified *Bacillota* members are obligate anaerobes of the *Clostridia* class, some of them being thiosulfate reducers of the genus *Dethiosulfatibacter*. We do not observe these obligate anaerobes in the BNO microbial community since they are unable to thrive in the predominantly aerobic conditions of the spring. Another group absent in the BNO microbial community and present in the Outokumpu microbial community is that of the *Actinobacteria* lineage. *Actinobacteria* account for 3% on the Outokumpu 1,820 m site and for 7% on the Outokumpu 2,300 m site. The classified members of *Actinobacteria* belong to three genera, namely *Rhodococcus*, *Microbacterium*, and *Dietzia*. These genera have the heterotrophic oxidative type of metabolism, and their presence in anaerobic environments is quite unusual. The authors assume that it could be explained either by mixing of deep-underground waters with residual drilling fluids or by generating oxygen in radiolysis processes sufficient to provide the conditions necessary for aerobes (13).

The BNO microbial community, unlike the Outokumpu and the Kidd Creek microbial communities, contains huge portions of members participating in the geochemical nitrogen cycle as well as organotrophs. The former are represented with two phyla, which are not observed in the Outokumpu and Kidd Creek microbial communities, namely *Nitrospirota* and *Planctomycetota*. They are all aerobic bacteria, except for the family *Brocadiaceae* containing anammox members. The latter are represented with *Myxococcota*, *Gemmatimonadota*, *Armatimonadota*, *Cyanobacteriota* aerobic members, missing in the Outokumpu and Kidd Creek microbial communities. They all possess the ability for oxidative phosphorylation. Moreover, *Cyanobacteriota* and *Myxococcota* phyla are represented with bacteria of predatory lifestyle.

The Shulgan-Tash “Cave Curd” specimen possesses high abundances of *Planctomycetota*, *Gamma*- and *Alphaproteobacteria* lineages (73), like the BNO site. Interestingly, the families of the “Cave Curd” site metabolically resemble those of the BNO site: ammonia-oxidizing bacteria of *Chromatiaceae* family (*Gammaproteobacteria*) of the “Cave Curd” site resemble ammonia-oxidizing bacteria of *Nitrosomonadaceae* (*Betaproteobacteria*) of the BNO site, while *Hyphomicrobiales* order (*Alphaproteobacteria*) of the “Cave Curd” site is also highly represented in the BNO site. The *Gemmataceae* family of the “Cave Curd” site is also a heterotrophic family of the *Planctomycetota* phylum, just like *Phycisphaeraceae*, which is highly represented in the BNO microbial community. Nitrite-oxidizing bacteria of the *Nitrospira* genus are represented in both environments in comparable quantities (~2%). In contrast to the BNO site, the Shulgan-Tash “Cave Curd” site is inhabited by a large proportion of *Acidobacteriota*, which is a typical group of cave environments, but has no representatives in the BNO microbial community. The absence of the *Acidobacteriota* representatives in the BNO site could be explained by the neutral pH of water of the granitic environment, while the karst caves (such as Shulgan-Tash) are formed in calcite rocks as a result of leaching of rocks by acidic waters.

On the basis of beta-diversity data, we can assume that the BNO microbial community is neither a typical deep granitic nor a karst cave microbial community, but rather a unique transitional microbial community formed as a result of action of unique geochemical conditions: the mixing of deep underground waters rich in volcanic gases with atmospheric oxygen. Its shared high abundance of *Alpha- and Gammaproteobacteria* with sites like Kidd Creek and Outokumpu connects it phylogenetically to the deep biosphere. The presence of populations of *Nitrospirota* and *Planctomycetota* is a hallmark of aerobic, nutrient-cycling environments, just like the Shulgan “Cave Curd” karst system. The functional similarity to the Shulgan-Tash "Cave Curd" microbial community – specifically the presence of ammonia-oxidizing (*Nitrosomonadaceae/Chromatiaceae*) and nitrite-oxidizing (*Nitrospira*) bacteria – confirms this aerobic link. The presence of oxygen in the BNO site prevents the establishment of the obligate anaerobes that dominate most deep granitic sites and enables the proliferation of aerobic nitrifying and heterotrophic bacteria. This gives the BNO site a functional profile similar to aerobic karst niches but without the typical karst taxa. However, the BNO site remains distinct from the karst cave environments due to its specific geochemistry. The neutral pH of the granitic aquifer water prevents the colonization by acid-tolerant groups that are typical of many karst caves, such as *Acidobacteriota*, and the specific blend of volcanic gases provides the growth of microbes, which are not typical for karst cave microbial communities.

Most likely, the BNO spring is a dynamic biogeochemical interface where volcanic gases mix with oxygen to create a gradient that supports a specialized microbial community specifically adapted to (i) oxidize reduced compounds (e.g., methane, iron, and hydrogen) aerobically, (ii) perform nitrification from ammonia/ammonium sources in the deep water, and (iii) thrive in a stable aerobic environment that is unusual for the deep subsurface.

Thus, the microbial community of the BNO represents a special ecological niche—a highly specialized ecosystem existing on the boundary between the deep anoxic biosphere and the aerobic surface. This niche with specific geochemical conditions (volcanic gases + O_2_ + neutral pH) attracts representatives of both worlds (aerobic taxa from karst-cave-like systems and chemolithotrophs from granitic systems), and that makes the BNO spring microbial community an interesting model system for studying the transition from anaerobic to aerobic ecosystems.

### Conclusion

The BNO spring microbial community has a complex composition, with lithotrophs, organotrophs, autotrophs, and predatory bacteria. The overall composition of the BNO spring microbial community suggests that there is an intensive process of nitrogen compound transformations, while the sulfur cycle is poorly represented in the BNO microbial community. Though the conditions in the BNO spring are mostly aerobic and the predominant part of the microbial community appears to be oxygen-respiring bacteria, a minor presence of nitrogen-fixing bacteria, nitrate-reducing bacteria, iron-reducing bacteria, and anammox bacteria demonstrates that there are zones of anoxic conditions in the BNO spring. We believe that gases of the crustal and volcanic origin (hydrogen, methane, and ammonia), as well as reduced iron compounds, provide the energy source while methane and carbon dioxide provide the carbon source for the primary production of the BNO spring microbial community.

A comparison of the BNO spring microbial community with the microbial communities of deep granite sites and karst caves shows that the BNO spring contains a microbial community that is not typical of either deep granitic sites or karst caves, but likely represents a transitional hybrid between them, formed by specific geochemical conditions. It shares common members with the deep granitic sites, since they utilize the common substrates; however, it lacks anaerobic representatives due to aerobic conditions. It also shares similar aerobic representatives and metabolic analogs with aerobic microbial communities of karst caves but lacks some lineages typical of low-pH karst cave waters. Thus, it should be considered as a unique transitional microbial community that developed upon contact of volcanic gas-rich waters with air, providing an important model for understanding the formation of microbial communities at the interface between anoxic subsurface and oxygenated surface environments.

## SUPPLEMENTAL MATERIAL

Supplemental material spectrum.02103-25-s0001.pdf Bacterial composition of deep granitic and karst cave sites; description of how these data were compiled; a geological slice of Mount Andyrchi showing the location of the Baksan Neutrino Observatory; a map of deep granitic and karst cave sites; distribution of secondary metabolite gene clusters across phyla; and tables showing gene distribution.

ASM does not own the copyrights to Supplemental Material that may be linked to, or accessed through, an article. The authors have granted ASM a non-exclusive, world-wide license to publish the Supplemental Material files. Please contact the corresponding author directly for reuse.

## References

[1] Magnabosco C, Lin L-H, Dong H, Bomberg M, Ghiorse W, Stan-Lotter H, Pedersen K, Kieft TL, van Heerden E, Onstott TC. 2018. The biomass and biodiversity of the continental subsurface. Nature Geosci 11:707–717. doi:10.1038/s41561-018-0221-6

[2] Soares A, Edwards A, An D, Bagnoud A, Bradley J, Barnhart E, Bomberg M, Budwill K, Caffrey SM, Fields M, Gralnick J, Kadnikov V, Momper L, Osburn M, Mu A, Moreau JW, Moser D, Purkamo L, Rassner SM, Sheik CS, Sherwood Lollar B, Toner BM, Voordouw G, Wouters K, Mitchell AC. 2023. A global perspective on bacterial diversity in the terrestrial deep subsurface. Microbiology (Reading, Engl) 169. doi:10.1099/mic.0.001172 PMC9993121 36748549

[3] Onstott TC, Ehlmann BL, Sapers H, Coleman M, Ivarsson M, Marlow JJ, Neubeck A, Niles P. 2019. Paleo-rock-hosted life on earth and the search on mars: a review and strategy for exploration. Astrobiology 19:1230–1262. doi:10.1089/ast.2018.1960 31237436 PMC6786346

[4] Escudero C, Amils R. 2023. Hard rock dark biosphere and habitability. Front Astron Space Sci 10. doi:10.3389/fspas.2023.1203845

[5] Dai X, Wang Y, Luo L, Pfiffner SM, Li G, Dong Z, Xu Z, Dong H, Huang L. 2021. Detection of the deep biosphere in metamorphic rocks from the Chinese continental scientific drilling. Geobiology 19:278–291. doi:10.1111/gbi.12430 33559972

[6] Moser DP, Gihring TM, Brockman FJ, Fredrickson JK, Balkwill DL, Dollhopf ME, Lollar BS, Pratt LM, Boice E, Southam G, Wanger G, Baker BJ, Pfiffner SM, Lin LH, Onstott TC. 2005. Desulfotomaculum and Methanobacterium spp. dominate a 4- to 5-kilometer-deep fault. Appl Environ Microbiol 71:8773–8783. doi:10.1128/AEM.71.12.8773-8783.2005 16332873 PMC1317344

[7] Purkamo L, Kietäväinen R, Nuppunen-Puputti M, Bomberg M, Cousins C. 2020. Ultradeep microbial communities at 4.4 km within crystalline bedrock: Implications for habitability in a planetary context. Life (Basel) 10:2. doi:10.3390/life10010002 31947979 PMC7175195

[8] Ruff SE, de Angelis IH, Mullis M, Payet JP, Magnabosco C, Lloyd KG, Sheik CS, Steen AD, Shipunova A, Morozov A, Reese BK, Bradley JA, Lemonnier C, Schrenk MO, Joye SB, Huber JA, Probst AJ, Morrison HG, Sogin ML, Ladau J, Colwell F. 2024. A global comparison of surface and subsurface microbiomes reveals large-scale biodiversity gradients, and a marine-terrestrial divide. Sci Adv 10:eadq0645. doi:10.1126/sciadv.adq0645 39693444 PMC11654699

[9] Magnabosco C, Ryan K, Lau MCY, Kuloyo O, Sherwood Lollar B, Kieft TL, van Heerden E, Onstott TC. 2016. A metagenomic window into carbon metabolism at 3 km depth in Precambrian continental crust. ISME J 10:730–741. doi:10.1038/ismej.2015.150 26325359 PMC4817676

[10] Lau MCY, Kieft TL, Kuloyo O, Linage-Alvarez B, van Heerden E, Lindsay MR, Magnabosco C, Wang W, Wiggins JB, Guo L, Perlman DH, Kyin S, Shwe HH, Harris RL, Oh Y, Yi MJ, Purtschert R, Slater GF, Ono S, Wei S, Li L, Sherwood Lollar B, Onstott TC. 2016. An oligotrophic deep-subsurface community dependent on syntrophy is dominated by sulfur-driven autotrophic denitrifiers. Proc Natl Acad Sci USA 113:E7927–E7936. doi:10.1073/pnas.1612244113 27872277 PMC5150411

[11] Lopez-Fernandez M, Åström M, Bertilsson S, Dopson M. 2018. Depth and dissolved organic carbon shape microbial communities in surface influenced but not ancient saline terrestrial aquifers. Front Microbiol 9:2880. doi:10.3389/fmicb.2018.02880 30538690 PMC6277548

[12] Osterholz H, Turner S, Alakangas LJ, Tullborg E-L, Dittmar T, Kalinowski BE, Dopson M. 2022. Terrigenous dissolved organic matter persists in the energy-limited deep groundwaters of the Fennoscandian Shield. Nat Commun 13:4837. doi:10.1038/s41467-022-32457-z 35977924 PMC9385861

[13] Purkamo L, Bomberg M, Kietäväinen R, Salavirta H, Nyyssönen M, Nuppunen-Puputti M, Ahonen L, Kukkonen I, Itävaara M. 2016. Microbial co-occurrence patterns in deep Precambrian bedrock fracture fluids. Biogeosciences 13:3091–3108. doi:10.5194/bg-13-3091-2016

[14] Nuppunen-Puputti M, Kietäväinen R, Raulio M, Soro A, Purkamo L, Kukkonen I, Bomberg M. 2022. Epilithic microbial community functionality in deep oligotrophic continental bedrock. Front Microbiol 13:826048. doi:10.3389/fmicb.2022.826048 35300483 PMC8921683

[15] Purkamo L, Bomberg M, Nyyssönen M, Kukkonen I, Ahonen L, Kietäväinen R, Itävaara M. 2013. Dissecting the deep biosphere: retrieving authentic microbial communities from packer-isolated deep crystalline bedrock fracture zones. FEMS Microbiol Ecol 85:324–337. doi:10.1111/1574-6941.12126 23560597

[16] Konno U, Kouduka M, Komatsu DD, Ishii K, Fukuda A, Tsunogai U, Ito K, Suzuki Y. 2013. Novel microbial populations in deep granitic groundwater from Grimsel Test Site, Switzerland. Microb Ecol 65:626–637. doi:10.1007/s00248-013-0184-5 23340500

[17] Ino K, Hernsdorf AW, Konno U, Kouduka M, Yanagawa K, Kato S, Sunamura M, Hirota A, Togo YS, Ito K, Fukuda A, Iwatsuki T, Mizuno T, Komatsu DD, Tsunogai U, Ishimura T, Amano Y, Thomas BC, Banfield JF, Suzuki Y. 2018. Ecological and genomic profiling of anaerobic methane-oxidizing archaea in a deep granitic environment. ISME J 12:31–47. doi:10.1038/ismej.2017.140 28885627 PMC5739000

[18] Ino K, Konno U, Kouduka M, Hirota A, Togo YS, Fukuda A, Komatsu D, Tsunogai U, Tanabe AS, Yamamoto S, Iwatsuki T, Mizuno T, Ito K, Suzuki Y. 2016. Deep microbial life in high‐quality granitic groundwater from geochemically and geographically distinct underground boreholes. Environ Microbiol Rep 8:285–294. doi:10.1111/1758-2229.12379 26743638

[19] Dutta A, Sar P, Sarkar J, Dutta Gupta S, Gupta A, Bose H, Mukherjee A, Roy S. 2019. Archaeal communities in deep terrestrial subsurface underneath the Deccan traps, India. Front Microbiol 10. doi:10.3389/fmicb.2019.01362 PMC6646420 31379755

[20] Dutta A, Dutta Gupta S, Gupta A, Sarkar J, Roy S, Mukherjee A, Sar P. 2018. Exploration of deep terrestrial subsurface microbiome in late Cretaceous Deccan traps and underlying Archean basement, India. Sci Rep 8. doi:10.1038/s41598-018-35940-0 PMC6265293 30498254

[21] Nazina TN, Luk’yanova EA, Zakharova EV, Konstantinova LI, Kalmykov SN, Poltaraus AB, Zubkov AA. 2010. Microorganisms in a disposal site for liquid radioactive wastes and their influence on radionuclides. Geomicrobiol J 27:473–486. doi:10.1080/01490451003719044

[22] Abramova E, Popova N, Artemiev G, Boldyrev K, Kazakov K, Kryuchkov D, Safonov A. 2023. Biological factors affecting the evolution of safety barrier materials in the Yeniseisky deep geological repository. Eng Geol 312:106931. doi:10.1016/j.enggeo.2022.106931

[23] Rampelotto PH. 2013. Extremophiles and extreme environments. Life (Basel) 3:482–485. doi:10.3390/life3030482 25369817 PMC4187170

[24] Anda D, Szabó A, Kovács-Bodor P, Makk J, Felföldi T, Ács É, Mádl-Szőnyi J, Borsodi AK. 2020. In situ modelling of biofilm formation in a hydrothermal spring cave. Sci Rep 10:21733. doi:10.1038/s41598-020-78759-4 33303927 PMC7729855

[25] Borsodi AK, Anda D, Makk J, Krett G, Dobosy P, Büki G, Erőss A, Mádl-Szőnyi J. 2018. Biofilm forming bacteria and archaea in thermal karst springs of Gellért Hill discharge area (Hungary). J Basic Microbiol 58:928–937. doi:10.1002/jobm.201800138 30160784

[26] Kadnikov VV, Mardanov AV, Beletsky AV, Karnachuk OV, Ravin NV. 2020. Microbial life in the deep subsurface aquifer illuminated by metagenomics. Front Microbiol 11:572252. doi:10.3389/fmicb.2020.572252 33013807 PMC7509429

[27] Nazina TN, Kosareva IM, Petrunyaka VV, Savushkina MK, Kudriavtsev EG, Lebedev VA, Ahunov VD, Revenko YA, Khafizov RR, Osipov GA, Belyaev SS, Ivanov MV. 2004. Microbiology of formation waters from the deep repository of liquid radioactive wastes Severnyi. FEMS Microbiol Ecol 49:97–107. doi:10.1016/j.femsec.2004.02.017 19712387

[28] Zavarzina DG, Maslov AA, Merkel AY, Kharitonova NA, Klyukina AA, Baranovskaya EI, Baydariko EA, Potapov EG, Zayulina KS, Bychkov AY, Chernyh NA, Bonch-Osmolovskaya EA, Gavrilov SN. 2025. Analogs of Precambrian microbial communities formed de novo in Caucasian mineral water aquifers. mBio 16:e0283124. doi:10.1128/mbio.02831-24 39660920 PMC11708057

[29] Milyukov VK, Myasnikov AV. 2023. A model for a new peripheral shallow magma chamber beneath the elbrus volcanic center. J Volcanolog Seismol 17:210–218. doi:10.1134/S0742046323700173

[30] Milyukov V, Rogozhin E, Gorbatikov A, Mironov A, Myasnikov A, Stepanova M. 2018. Contemporary state of the Elbrus volcanic center (The Northern Caucasus). Pure Appl Geophys 175:1889–1907. doi:10.1007/s00024-017-1595-x

[31] Pershin SM, Sobisevich AL, Makarov VS, Myasnikov AV, Grishin MYa, Zavozin VA, Lednev VN, Likhodeev DV, Kazalov VV. 2023. Lidar monitoring of magmatic activity in the small chamber of the Elbrus volcanic center. Dokl Phys 68:72–76. doi:10.1134/S1028335823030047

[32] Tarasov K, Kravchenko E, Zarubin M, Yakhnenko A. 2024. Deep underground metagenome-assembled genomes from hydrothermal spring. Microbiol Resour Announc 13:e0057424. doi:10.1128/mra.00574-24 39283138 PMC11465946

[33] Andrews KT, Gupta AP, Tran TN, Fairlie DP, Gobert GN, Bozdech Z. 2012. Comparative gene expression profiling of P. falciparum malaria parasites exposed to three different histone deacetylase inhibitors. PLoS One 7:e31847. doi:10.1371/journal.pone.0031847 22384084 PMC3288058

[34] Ewels P, Magnusson M, Lundin S, Käller M. 2016. MultiQC: summarize analysis results for multiple tools and samples in a single report. Bioinformatics 32:3047–3048. doi:10.1093/bioinformatics/btw354 27312411 PMC5039924

[35] Nurk S, Meleshko D, Korobeynikov A, Pevzner PA. 2017. metaSPAdes: a new versatile metagenomic assembler. Genome Res 27:824–834. doi:10.1101/gr.213959.116 28298430 PMC5411777

[36] Kang DD, Li F, Kirton E, Thomas A, Egan R, An H, Wang Z. 2019. MetaBAT 2: an adaptive binning algorithm for robust and efficient genome reconstruction from metagenome assemblies. PeerJ 7:e7359. doi:10.7717/peerj.7359 31388474 PMC6662567

[37] Parks DH, Imelfort M, Skennerton CT, Hugenholtz P, Tyson GW. 2015. CheckM: assessing the quality of microbial genomes recovered from isolates, single cells, and metagenomes. Genome Res 25:1043–1055. doi:10.1101/gr.186072.114 25977477 PMC4484387

[38] Chaumeil PA, Mussig AJ, Hugenholtz P, Parks DH. 2022. GTDB-Tk v2: memory friendly classification with the genome taxonomy database. Bioinformatics 38:5315–5316. doi:10.1093/bioinformatics/btac672 36218463 PMC9710552

[39] Chakoory O, Comtet-Marre S, Peyret P. 2022. RiboTaxa: combined approaches for rRNA genes taxonomic resolution down to the species level from metagenomics data revealing novelties. NAR Genom Bioinform 4:lqac070. doi:10.1093/nargab/lqac070 36159175 PMC9492272

[40] Zhou Z, Tran PQ, Breister AM, Liu Y, Kieft K, Cowley ES, Karaoz U, Anantharaman K. 2022. METABOLIC: high-throughput profiling of microbial genomes for functional traits, metabolism, biogeochemistry, and community-scale functional networks. Microbiome 10:33. doi:10.1186/s40168-021-01213-8 35172890 PMC8851854

[41] Zheng J, Ge Q, Yan Y, Zhang X, Huang L, Yin Y. 2023. dbCAN3: automated carbohydrate-active enzyme and substrate annotation. Nucleic Acids Res 51:W115–W121. doi:10.1093/nar/gkad328 37125649 PMC10320055

[42] Garber AI, Nealson KH, Okamoto A, McAllister SM, Chan CS, Barco RA, Merino N. 2020. FeGenie: a comprehensive tool for the identification of iron genes and iron gene neighborhoods in genome and metagenome assemblies. Front Microbiol 11:37. doi:10.3389/fmicb.2020.00037 32082281 PMC7005843

[43] Blin K, Shaw S, Augustijn HE, Reitz ZL, Biermann F, Alanjary M, Fetter A, Terlouw BR, Metcalf WW, Helfrich EJN, van Wezel GP, Medema MH, Weber T. 2023. antiSMASH 7.0: new and improved predictions for detection, regulation, chemical structures and visualisation. Nucleic Acids Res 51:W46–W50. doi:10.1093/nar/gkad344 37140036 PMC10320115

[44] Pritchard L, Glover RH, Humphris S, Elphinstone JG, Toth IK. 2016. Genomics and taxonomy in diagnostics for food security: soft-rotting enterobacterial plant pathogens. Anal Methods 8:12–24. doi:10.1039/C5AY02550H

[45] Huerta-Cepas J, Serra F, Bork P. 2016. ETE 3: reconstruction, analysis, and visualization of phylogenomic data. Mol Biol Evol 33:1635–1638. doi:10.1093/molbev/msw046 26921390 PMC4868116

[46] Pershin SM, Gordeev EI, Grishin My, Zavozin VA, Makarov VS, Lednev VN, Ponurovskii Y, Stavrovskii DB, Ushakov AA, Kazalov VV. 2024. Monitoring the baric modulation of gas concentration in the Baksan Neutrino Observatory tunnel in the elbrus region using differential absorption lidar. Dokl Earth Sc 515:535–540. doi:10.1134/S1028334X23603164

[47] Aydarkozhina AS, Lavrushin Vy, Ermakov AV, Chelnokov GA, Zhang L. 2024. СO2-rich thermal waters of the Neutrino research tunnel (Baksan Neutrino Observatory, North Caucasus). Dokl Earth Sc 515:526–534. doi:10.1134/S1028334X23603334

[48] Roy C, Rameez MJ, Haldar PK, Peketi A, Mondal N, Bakshi U, Mapder T, Pyne P, Fernandes S, Bhattacharya S, Roy R, Mandal S, O’Neill WK, Mazumdar A, Mukhopadhyay SK, Mukherjee A, Chakraborty R, Hallsworth JE, Ghosh W. 2020. Microbiome and ecology of a hot spring-microbialite system on the Trans-Himalayan Plateau. Sci Rep 10:5917. doi:10.1038/s41598-020-62797-z 32246033 PMC7125080

[49] Stout LM, Blake RE, Greenwood JP, Martini AM, Rose EC. 2009. Microbial diversity of boron-rich volcanic hot springs of St. Lucia, Lesser Antilles. FEMS Microbiol Ecol 70:402–412. doi:10.1111/j.1574-6941.2009.00780.x 19796138

[50] Bowers RM, Kyrpides NC, Stepanauskas R, Harmon-Smith M, Doud D, Reddy TBK, Schulz F, Jarett J, Rivers AR, Eloe-Fadrosh EA, et al.. 2017. Minimum information about a single amplified genome (MISAG) and a metagenome-assembled genome (MIMAG) of bacteria and archaea. Nat Biotechnol 35:725–731. doi:10.1038/nbt.3893 28787424 PMC6436528

[51] Wang G, Tang M, Li T, Dai S, Wu H, Chen C, He H, Fan J, Xiang W, Li X. 2015. Wenzhouxiangella marina gen. nov, sp. nov, a marine bacterium from the culture broth of Picochlorum sp. 122, and proposal of Wenzhouxiangellaceae fam. nov. in the order Chromatiales. Antonie Van Leeuwenhoek 107:1625–1632. doi:10.1007/s10482-015-0458-7 25903846

[52] Sorokin DY, Mosier D, Zorz JK, Dong X, Strous M. 2020. Wenzhouxiangella strain AB-CW3, a proteolytic bacterium from hypersaline soda lakes that preys on cells of Gram-positive bacteria. Front Microbiol 11:597686. doi:10.3389/fmicb.2020.597686 33281797 PMC7691419

[53] Bodrossy L, Holmes EM, Holmes AJ, Kovács KL, Murrell JC. 1997. Analysis of 16S rRNA and methane monooxygenase gene sequences reveals a novel group of thermotolerant and thermophilic methanotrophs, Methylocaldum gen. nov. Arch Microbiol 168:493–503. doi:10.1007/s002030050527 9385141

[54] Takeuchi M, Kamagata Y, Oshima K, Hanada S, Tamaki H, Marumo K, Maeda H, Nedachi M, Hattori M, Iwasaki W, Sakata S. 2014. Methylocaldum marinum sp. nov., a thermotolerant, methane-oxidizing bacterium isolated from marine sediments, and emended description of the genus methylocaldum. Int J Syst Evol Microbiol 64:3240–3246. doi:10.1099/ijs.0.063503-0 24981325

[55] Diederen BMW. 2008. Legionella spp. and Legionnaires’ disease. J Infect 56:1–12. doi:10.1016/j.jinf.2007.09.010 17980914

[56] Rosenberg E, DeLong EF, Lory S, Stackebrandt E, Thompson F. 2013. The prokaryotes: Gammaproteobacteria. Springer Berlin Heidelberg.

[57] Vignais PM, Billoud B. 2007. Occurrence, classification, and biological function of hydrogenases: an overview. Chem Rev 107:4206–4272. doi:10.1021/cr050196r 17927159

[58] Shin YH, Kim JH, Suckhoom A, Kantachote D, Kim W. 2017. Limibaculum halophilum gen. nov., sp. nov., a new member of the family Rhodobacteraceae. Int J Syst Evol Microbiol 67:3812–3818. doi:10.1099/ijsem.0.002200 28879850

[59] Rosenberg E. 2013. The prokaryotes: Alphaproteobacteria and Betaproteobacteria. Springer.

[60] Rosenberg E, Long EF, Lory S, Stackebrandt E, Thompson F. 2014. The prokaryotes: other major lineages of bacteria and the archaea. Springer.

[61] Zhang L, Dong T, Yang J, Hao S, Sun Z, Peng Y. 2023. Anammox coupled with photocatalyst for enhanced nitrogen removal and the activated aerobic respiration of anammox bacteria based on cbb3-type cytochrome c oxidase. Environ Sci Technol 57:17910–17919. doi:10.1021/acs.est.3c02435 37463493

[62] van Kessel M, Speth DR, Albertsen M, Nielsen PH, Op den Camp HJM, Kartal B, Jetten MSM, Lücker S. 2015. Complete nitrification by a single microorganism. Nature 528:555–559. doi:10.1038/nature16459 26610025 PMC4878690

[63] Garcia R, Müller R. 2014. The family polyangiaceae, p 247–279. *In* The prokaryotes: deltaproteobacteria and epsilonproteobacteria. Springer Berlin Heidelberg.

[64] Garcia R, Müller R. 2013. The family myxococcaceae, p 191–212. *In* The prokaryotes: deltaproteobacteria and epsilonproteobacteria. Springer Berlin Heidelberg.

[65] Pandelia ME, Lubitz W, Nitschke W. 2012. Evolution and diversification of Group 1 [NiFe] hydrogenases. Is there a phylogenetic marker for O(2)-tolerance? Biochim Biophys Acta 1817:1565–1575. doi:10.1016/j.bbabio.2012.04.012 22588050

[66] Gong X, Xu L, Langwig MV, Chen Z, Huang S, Zhao D, Su L, Zhang Y, Francis CA, Liu J, Li J, Baker BJ. 2024. Globally distributed marine Gemmatimonadota have unique genomic potentials. Microbiome 12. doi:10.1186/s40168-024-01871-4 PMC11316326 39123272

[67] Jurkevitch E. 2007. Predatory prokaryotes. 1st ed. Springer.

[68] Bankar A, Patil S, Shinde M, Shinde S, Kowligi B. 2021. Potential of microbial extremophiles for biotechnological applications: an overview, p 89–109. *In* Microbial extremozymes: novel sources and industrial applications. Patricia Osborn (Elsevier).

[69] Bowman J. 2006. The methanotrophs — the families Methylococcaceae and Methylocystaceae, p 266–289. In The prokaryotes. Springer New York.

[70] Wilpiszeski RL, Sherwood Lollar B, Warr O, House CH. 2020. In situ growth of halophilic bacteria in saline fracture fluids from 2.4 km below surface in the deep canadian shield. Life (Basel) 10:307. doi:10.3390/life10120307 33255232 PMC7760289

[71] Deng X, Dohmae N, Nealson KH, Hashimoto K, Okamoto A. 2018. Multi-heme cytochromes provide a pathway for survival in energy-limited environments. Sci Adv 4:eaao5682. doi:10.1126/sciadv.aao5682 29464208 PMC5815863

[72] Payler SJ, Biddle JF, Sherwood Lollar B, Fox-Powell MG, Edwards T, Ngwenya BT, Paling SM, Cockell CS. 2019. An ionic limit to life in the deep subsurface. Front Microbiol 10:426. doi:10.3389/fmicb.2019.00426 30915051 PMC6422919

[73] Gogoleva N, Chervyatsova O, Balkin A, Kuzmina L, Shagimardanova E, Kiseleva D, Gogolev Y. 2023. Microbial tapestry of the Shulgan-Tash cave (Southern Ural, Russia): influences of environmental factors on the taxonomic composition of the cave biofilms. Environ Microbiome 18:82. doi:10.1186/s40793-023-00538-1 37990336 PMC10662634

