# Supplementary material for: Metagenomic analysis of the biofilm community at the oxic-anoxic interface of a deep-underground saline spring at the Baksan Neutrino Observatory
Source: Microbiol Spectr. 2026 Feb 27;14(4):e02103-25. doi: 10.1128/spectrum.02103-25 (PMC13055281; doi:10.1128/spectrum.02103-25)
Supplement: Supplemental material — Bacterial composition of deep granitic and karst cave sites; description of how these data were compiled; a geological slice of Mount Andyrchi showing the location of the Baksan Neutrino Observatory; a map of deep granitic and karst cave sites; distribution of secondary metabolite gene clusters across phyla; and tables showing gene distribution. [file spectrum.02103-25-s0001.pdf]

Supplemental Materials

Supplementary Table 1. Abundances of bacterial phyla in deep granitic environments. The *Pseudomonadota* phylum is represented on the class level with 4 classes (*Alpha-, Beta-, Gamma-*, and *Deltaproteobacteria*) while *Archaea* are represented on the domain level. The cutoff value of the abundance for each group was selected 0.2%, the groups sharing the abundance in the metagenome less than this value were added to the group “Other”. The BNO SSU column shows the abundances of bacterial lineages of the spring of the Baksan Neutrino Observatory obtained with the RiboTaxa pipeline, while the BNO column features the abundances derived from the MAG classification using the GTDB-tk tool. Supplementary Table 2 describes the way the data were compiled.

Phylum	BNO	BNO SSU	Boulby	Nevada average	Chixulub	Deccan Traps	SURF D1
Alphaproteobacteria	7.86	13.52	12.75	10.6	14.7	7	3
Betaproteobacteria	22.83	24.4	0.2			6.9	22.5
Gammaproteobacteria	43.23	37.04	11.03	32.21	52.4	28.4	3.8
Campylobacterota							
Planctomycetota	16.02	15.01		1.26	1	1	1.2
Myxococcota	3.08			0.52			
Nitrospirota	2.55	1.66		2.44			2.8
Gemmatimonadota	1.74	0.99					
Armatimonadota	1.45	1.02		0.43			
Cyanobacteriota	1.24	1.26				1.3	
Chloroflexota		1.5		2.98		2	1.3
Firmicutes			2.15	15.41	3.6	10	0.8
Chlorobiota							
Bacteroidota			2.2	15.7	8.9	2.9	1.3
Acidobacteriota				1.34	3	1.5	
Thermodesulfobacterota			4.27	0.87			3.4
Actinobacteriota				1.1	5.6	31.6	0.5
Bdellovibrionota				1.42	2.4		
Deferribacterota							
Candidate OD1							
Candidate OP11							
Candidate OP3							
Deinococcota				1.99	3		
Candidate Latescibacterota							
Spirochaetota				0.42			0.9
Mycoplasmatota (Tenericutes)							
Omnitrophicaeota							20.4
Bipolaricaulota							
Patescibacterota (CPR group)		0.05		0.7			7.8
Candidate Zixibacterota							0.8
Candidate Methylomirabilota							
Candidate Rokuibacteriota							
Verrucomicrobiota				1.11			
Dadabacteriota		0.84					
Fusobacteriota							
Atribacterota							
Candidate TM6							
Elusimicrobiota				0.27			
DTB-120		0.39		0.22			
MBNT15				0.71			
WPS-2				0.24			
Archaea		0.13	66.75	6.04		1.7	3
Fungi							
UBP18							
Unclassified						1.4	26.5
Other		2.19	0.65	2.02	5.4	4.3	
Total	0	0	0	0	0	0	0
Depth	1700	1700	1100	407	1035	408	244
Temperature	42	42	30	32.3	57.9	-	10.3

Phylum	SURF D2	SURF D3	SURF D4	SURF D5	SURF D6	Aspo MM	Aspo OS
Alphaproteobacteria	0.6	1.4	4.5	1.5	0.8	5.03	4.23
Betaproteobacteria	28.4	17	26.7	21.9	0.4		
Gammaproteobacteria	7.4	2.7	10	10.8		30.18	10.71
Campylobacterota						21.53	31.39
Planctomycetota	1.7	1.7	0.8	0.8			
Myxococcota							
Nitrospirota	3.5	11.9	3.6	4.3	0.6	0.62	0.87
Gemmatimonadota							
Armatimonadota							
Cyanobacteriota							
Chloroflexota	1.5	4.8	1.1	1.8	3.4	2.49	11.59
Firmicutes		0.8	2.5	8.6	27.2	1.18	1.55
Chlorobiota							
Bacteroidota	6.4	5.2	5.2	3.6	10.8	0.56	0.9
Acidobacteriota						0.57	2.69
Thermodesulfobacterota	11.3	12.2	4	12.6	32.2	11.16	10.14
Actinobacteriota	0.5	1.4	1.8	4.5		0.95	3.48
Bdellovibrionota							
Deferribacterota							
Candidate OD1							
Candidate OP11							
Candidate OP3							
Deinococcota							
Candidate Latescibacterota							
Spirochaetota	3.4	2.4	0.9	1		0.49	0.37
Mycoplasmatota (Tenericutes)					2.4	0.47	0.36
Omnitrophicaeota	8.1	2.8	13.2	8.1	4.1	0.88	0.9
Bipolaricaulota					8.2		
Patescibacterota (CPR group)	7.3	3.8	3.6	6	2.6	20.72	12.57
Candidate Zixibacterota	1	5					
Candidate Methylomirabilota							
Candidate Rokuibacteriota			0.5	1	1.8		
Verrucomicrobiota							
Dadabacteriota							
Fusobacteriota							
Atribacterota						1.75	6.31
Candidate TM6							
Elusimicrobiota							
DTB-120							
MBNT15							
WPS-2							
Archaea	4.4	8.2	12.9	3		0.27	1.12
Fungi							
UBP18							
Unclassified	14.5	18.7	8.7	10.5	5.5		
Other						1.15	0.82
Total	0	0	0	0	0	0	0
Depth	244	610	1250	1478	1478	331	310.6
Temperature	12.5	16.2	22.6	31.8	21.6	-	-

Phylum	Aspo TM	Olkiluoto	Outokumpu 180m	Outokumpu 500m	Outokumpu 967m
Alphaproteobacteria	57.9	2.99		10.31	3.09
Betaproteobacteria		0.55	75.26	74.23	7.22
Gammaproteobacteria	17.9	0.26		2.06	
Campylobacterota	0.94	0.04			
Planctomycetota					
Myxococcota	0.72				
Nitrospirota	0.24				
Gemmatimonadota					
Armatimonadota					
Cyanobacteriota					
Chloroflexota	4.34	0.21	1.03		
Firmicutes		43.9	2.06	5.15	55.67
Chlorobiota					
Bacteroidota	2.05	1.31	13.4		8.25
Acidobacteriota	0.91				
Thermodesulfobacterota	7.97	38.32			
Actinobacteriota	0.5	0.12		1.03	4.12
Bdellovibrionota					
Deferribacterota					
Candidate OD1		0.26	5.15		
Candidate OP11					
Candidate OP3					
Deinococcota					
Candidate Latescibacterota					
Spirochaetota	1.12	0.01	1.03		
Mycoplasmatota (Tenericutes)		3.78	1.03	6.19	11.34
Omnitrophicaeota					
Bipolaricaulota					
Patescibacterota (CPR group)	1.71				
Candidate Zixibacterota	0.63				
Candidate Methylomirabilota					
Candidate Rokuibacteriota					
Verrucomicrobiota					
Dadabacteriota					
Fusobacteriota					
Atribacterota	2.22				
Candidate TM6		0.41			
Elusimicrobiota		0.09			
DTB-120					
MBNT15					
WPS-2					
Archaea	0.12	7.08			
Fungi					
UBP18					
Unclassified			1.04	1.03	10.31
Other	0.73	0.67			
Total	0	0	0	0	0
Depth	448.4	530	180	500	967
Temperature	-	12.6	-	-	-

Phylum	Outokumpu 1820m	Outokumpu 2260m	Outokumpu 2300m	Pyhäsalmi	Otaniemi	Grimsel
Alphaproteobacteria	3.12		2.11	20.63	4.23	12.03
Betaproteobacteria	22.92	33.51	32.63			12.94
Gammaproteobacteria	32.29	5.08	30.53	77.4	55.41	
Campylobacterota						
Planctomycetota					0.63	0.21
Myxococcota						
Nitrospirota						
Gemmatimonadota						
Armatimonadota						
Cyanobacteriota				0.12	0.84	0.86
Chloroflexota				0.07		0.2
Firmicutes	28.12	4.06	22.11		10.72	51.22
Chlorobiota						
Bacteroidota				0.23	7.1	5.11
Acidobacteriota					0.59	0.43
Thermodesulfobacterota						13.96
Actinobacteriota	3.12	53.82	7.37		12.11	0.15
Bdellovibrionota						
Deferribacterota						
Candidate OD1						
Candidate OP11						
Candidate OP3						
Deinococcota	1.04					
Candidate Latescibacterota						
Spirochaetota						2.39
Mycoplasmatota (Tenericutes)						
Omnitrophicaeota						
Bipolaricaulota						
Patescibacterota (CPR group)						
Candidate Zixibacterota						
Candidate Methylomirabilota						
Candidate Rokuibacteriota						
Verrucomicrobiota				0.35		0.32
Dadabacteriota						0.14
Fusobacteriota					0.11	
Atribacterota						
Candidate TM6						
Elusimicrobiota						
DTB-120						
MBNT15						
WPS-2						
Archaea		2.51				
Fungi						
UBP18						
Unclassified	9.39	1.02	5.25	1.08	8.26	
Other				0.12		0.04
Total	0	0	0	0	0	0
Depth	1820	2260	2300	2400	3927	450
Temperature	-	-	-	23	61	-

Phylum	Kidd Creek FW12299 slide	Kidd Creek FW12299 filter	Kidd Creek FW12322 slide
Alphaproteobacteria	2.28	36.03	1.67
Betaproteobacteria	26.02	63.67	22.1
Gammaproteobacteria	12.27		40.14
Campylobacterota			
Planctomycetota			
Myxococcota			
Nitrospirota			
Gemmatimonadota			
Armatimonadota			
Cyanobacteriota			
Chloroflexota			
Firmicutes	57.08	0.07	8.33
Chlorobiota			
Bacteroidota	1.09		27.14
Acidobacteriota			
Thermodesulfobacterota			
Actinobacteriota	1.15	0.22	0.51
Bdellovibrionota			
Deferribacterota			
Candidate OD1			
Candidate OP11			
Candidate OP3			
Deinococcota			
Candidate Latescibacterota			
Spirochaetota			
Mycoplasmatota (Tenericutes)			
Omnitrophicaeota			
Bipolaricaulota			
Patescibacterota (CPR group)			
Candidate Zixibacterota			
Candidate Methylomirabilota			
Candidate Rokuibacteriota			
Verrucomicrobiota			0.05
Dadabacteriota			
Fusobacteriota			
Atribacterota			
Candidate TM6			
Elusimicrobiota			
DTB-120			
MBNT15			
WPS-2			
Archaea			
Fungi			
UBP18			
Unclassified	0.11	0.01	0.06
Other			
Total	0	0	0
Depth	2400	2400	2400
Temperature	26	26	25

Phylum	Kidd Creek FW12287 A filter	Mizunami	Dobrovolnoye R-224	Dobrovolnoye G-15	Severnyi D-1
Alphaproteobacteria	9.8	9.94	5.4	32.1	1.53
Betaproteobacteria	16.85	16.83			
Gammaproteobacteria	45.69	1.72	14	59	
Campylobacterota					
Planctomycetota	0.81				
Myxococcota					
Nitrospirota		16.25			
Gemmatimonadota		0.57			
Armatimonadota					
Cyanobacteriota			2.9		
Chloroflexota		0.76			
Firmicutes	18.8	13.58	24.7	6.5	3.51
Chlorobiota		9.94			
Bacteroidota	3.86	6.5	9.3	1	
Acidobacteriota				1.4	
Thermodesulfobacterota		15.11			
Actinobacteriota	1.8	1.34	15.8		77.28
Bdellovibrionota					
Deferribacterota					
Candidate OD1					
Candidate OP11					
Candidate OP3					
Deinococcota	0.66		0.4		
Candidate Latescibacterota					
Spirochaetota		0.38	4		
Mycoplasmatota (Tenericutes)					
Omnitrophicaeota					
Bipolaricaulota					
Patescibacterota (CPR group)	0.48				
Candidate Zixibacterota					
Candidate Methylomirabilota					
Candidate Rokuibacteriota					
Verrucomicrobiota		0.19			
Dadabacteriota					
Fusobacteriota			23.5		
Atribacterota					
Candidate TM6					
Elusimicrobiota					
DTB-120					
MBNT15					
WPS-2					
Archaea	0.33	4.21			
Fungi					17.68
UBP18					
Unclassified	0.92				
Other		2.68			
Total	0	0	0	0	0
Depth	2400	300			230
Temperature	25.1	23	-	-	-

Phylum	Soudan	Witwatersrand Be326	Witwatersrand Dr5IPC	Witwatersrand FI88
Alphaproteobacteria	19.67	9.8	5.4	5.9
Betaproteobacteria		18.85	12.4	15.4
Gammaproteobacteria	12	16.55	10.8	9
Campylobacterota		0.45	1	1
Planctomycetota		1.05	2.7	2.2
Myxococcota				
Nitrospirota		2.6	2.6	3.2
Gemmatimonadota				
Armatimonadota				
Cyanobacteriota		0.1	0.2	0.5
Chloroflexota		4.8	7.1	5.8
Firmicutes	26.11	8.35	4.8	4.9
Chlorobiota		1.7	4.6	3.2
Bacteroidota	0.44	3.35	3.2	3.4
Acidobacteriota		1.4	1.4	1.6
Thermodesulfobacterota	10.78	9.35	12.3	20.4
Actinobacteriota	2.22	2.5	2.4	3
Bdellovibrionota				
Deferribacterota		1.05	1.9	1.4
Candidate OD1		1.45	1.3	0.7
Candidate OP11				
Candidate OP3		0.15	0.7	0.7
Deinococcota		0.9	1.3	1.3
Candidate Latescibacterota				
Spirochaetota	0.11			
Mycoplasmatota (Tenericutes)				
Omnitrophicaeota				
Bipolaricaulota				
Patescibacterota (CPR group)				
Candidate Zixibacterota				
Candidate Methylomirabilota				
Candidate Rokuibacteriota				
Verrucomicrobiota		0.1	0.2	0.5
Dadabacteriota				
Fusobacteriota		0.1		
Atribacterota		0.25	1.5	0.8
Candidate TM6				
Elusimicrobiota				
DTB-120				
MBNT15				
WPS-2				
Archaea	12.56			
Fungi				
UBP18	0.11			
Unclassified	16	9.8	15.1	9.3
Other		5.35	7.1	5.8
Total	0	0	0	0
Depth	700	1339	1046	1056
Temperature	-	37.5	26.8	28.6

Phylum	Witwatersrand MM5194	Witwatersrand NO14	Witwatersrand TT109	Borra caves
Alphaproteobacteria	8.2	8.8	5.5	24.6
Betaproteobacteria	14	15.3	18.2	7.3
Gammaproteobacteria	15.7	19.5	13.7	23
Campylobacterota	0.7	1	1	
Planctomycetota	2.4	1.8	1.5	
Myxococcota				
Nitrospirota	2.7	2.4	2.2	
Gemmatimonadota				
Armatimonadota				
Cyanobacteriota	0.3	0.5	0.3	
Chloroflexota	6.3	5.7	6	
Firmicutes	5.5	5.8	5.2	2.6
Chlorobiota	1.2	1	3.7	
Bacteroidota	4	3.8	3.5	5
Acidobacteriota	1.3	1.4	1.3	
Thermodesulfobacterota	9.2	9.4	11.2	
Actinobacteriota	2.5	2.6	2	29
Bdellovibrionota				
Deferribacterota	1.2	1.2	1.1	
Candidate OD1	2.5	2.3	1.1	
Candidate OP11				
Candidate OP3	0.7	0.8	0.8	
Deinococcota	0.6	0.8	4.5	
Candidate Latescibacterota				
Spirochaetota				
Mycoplasmatota (Tenericutes)				
Omnitrophicaeota				
Bipolaricaulota				
Patescibacterota (CPR group)				
Candidate Zixibacterota				
Candidate Methylomirabilota				
Candidate Rokuibacteriota				
Verrucomicrobiota	0.3	0.6	0.3	
Dadabacteriota				
Fusobacteriota	0.3		0.3	
Atribacterota	1.8	1.2	0.9	
Candidate TM6				
Elusimicrobiota				
DTB-120				
MBNT15				
WPS-2				
Archaea				
Fungi				
UBP18				
Unclassified	11.2	7.8	9.3	
Other	7.4	6.3	6.4	8.5
Total	0	0	0	
Depth	1900	2100	3136	
Temperature	40.7	65	48.7	

Phylum	Yumugi	Grotta grande C1	Grotta grande C2	Ovito di Pietrasecca C3
Alphaproteobacteria	19.7	7.8	3.1	4.2
Betaproteobacteria	4.4			
Gammaproteobacteria	16.1	26.9	17.2	20
Campylobacterota				
Planctomycetota		9.2	14.2	16.8
Myxococcota				
Nitrospirota	1.2			1.9
Gemmatimonadota			4.2	
Armatimonadota				
Cyanobacteriota				
Chloroflexota	1.7	11.4	15.8	9.6
Firmicutes	1.9	12.8	10.8	
Chlorobiota		6.1		8.6
Bacteroidota				
Acidobacteriota	2	13.6	23	19.7
Thermodesulfobacterota				
Actinobacteriota	40.3	12.2	7.5	3.9
Bdellovibrionota				
Deferribacterota				
Candidate OD1				
Candidate OP11				
Candidate OP3				
Deinococcota				
Candidate Latescibacterota				
Spirochaetota				
Mycoplasmatota (Tenericutes)				
Omnitrophicaeota				
Bipolaricaulota				
Patescibacterota (CPR group)				11.9
Candidate Zixibacterota				
Candidate Methylomirabilota			4.2	3.4
Candidate Rokuibacteriota				
Verrucomicrobiota				
Dadabacteriota				
Fusobacteriota				
Atribacterota				
Candidate TM6				
Elusimicrobiota				
DTB-120				
MBNT15				
WPS-2				
Archaea				
Fungi				
UBP18				
Unclassified	7			
Other	5.7			
Total	0	0	0	0
Depth				
Temperature				

Phylum	Ovito di Pietrasecca C4	Shulgan-Tash Azurite	Shulgan-Tash BrownWhite
Alphaproteobacteria	4.4	5.9	5.7
Betaproteobacteria			
Gammaproteobacteria	32	7.8	9.5
Campylobacterota			
Planctomycetota	18.2	2.8	5.2
Myxococcota		1	1.7
Nitrospirota	3.4	0.7	1.4
Gemmatimonadota			
Armatimonadota			
Cyanobacteriota			
Chloroflexota	13.4	1.1	3.3
Firmicutes	6.7		
Chlorobiota			
Bacteroidota		1.2	2.3
Acidobacteriota	21.9	3.6	6.3
Thermodesulfobacterota			
Actinobacteriota		72.2	57.7
Bdellovibrionota			
Deferribacterota			
Candidate OD1			
Candidate OP11			
Candidate OP3			
Deinococcota			
Candidate Latescibacterota			1.1
Spirochaetota			
Mycoplasmatota (Tenericutes)			
Omnitrophicaeota			
Bipolaricaulota			
Patescibacterota (CPR group)		0.7	
Candidate Zixibacterota			
Candidate Methylomirabilota			
Candidate Rokuibacteriota			
Verrucomicrobiota		0.5	1
Dadabacteriota		0.8	
Fusobacteriota			
Atribacterota			
Candidate TM6			
Elusimicrobiota			
DTB-120			
MBNT15			
WPS-2			
Archaea			0.6
Fungi			
UBP18			
Unclassified			
Other		1.7	4.2
Total	0	0	0
Depth			
Temperature			

Phylum	Shulgan-Tash Coralline	Shulgan-Tash Gray1Floor	Shulgan-Tash WhiteRhizoidFlat
Alphaproteobacteria	3.5	7.8	5.6
Betaproteobacteria			
Gammaproteobacteria	1.8	9.6	25.5
Campylobacterota			
Planctomycetota	1	4.6	4.2
Myxococcota		0.8	
Nitrospirota		1.2	0.5
Gemmatimonadota			0.9
Armatimonadota		0.3	0.8
Cyanobacteriota			
Chloroflexota	1.7	1.7	4.1
Firmicutes			
Chlorobiota			
Bacteroidota		1.2	
Acidobacteriota	1.7	7.8	6.1
Thermodesulfobacterota			
Actinobacteriota	89.8	59.3	42.8
Bdellovibrionota			
Deferribacterota			
Candidate OD1			
Candidate OP11			
Candidate OP3			
Deinococcota			
Candidate Latescibacterota			1.1
Spirochaetota			
Mycoplasmatota (Tenericutes)			
Omnitrophicaeota			
Bipolaricaulota			
Patescibacterota (CPR group)			
Candidate Zixibacterota			
Candidate Methylomirabilota		1	2.9
Candidate Rokuibacteriota			
Verrucomicrobiota		1.4	0.5
Dadabacteriota			
Fusobacteriota			
Atribacterota			
Candidate TM6			
Elusimicrobiota			
DTB-120			
MBNT15			
WPS-2			
Archaea			2.5
Fungi			
UBP18			
Unclassified			
Other	0.5	3.3	2.5
Total	0	0	0
Depth			
Temperature			

Phylum	Shulgan-Tash WhiteWavyEdge	Shulgan-Tash WhiteYellowEdge	Shulgan-Tash CaveCurd
Alphaproteobacteria	4.8	9.2	13.8
Betaproteobacteria			
Gammaproteobacteria	5	6.8	32.6
Campylobacterota			
Planctomycetota	6.6	7.5	16.7
Myxococcota	0.8	2.2	
Nitrospirota	1.5	1.2	1.4
Gemmatimonadota	0.8	0.8	
Armatimonadota	0.3	0.4	
Cyanobacteriota			1.4
Chloroflexota	3.5	2.9	3.6
Firmicutes			
Chlorobiota			
Bacteroidota	0.4	1.5	0.4
Acidobacteriota	8.2	7.5	17.3
Thermodesulfobacterota			
Actinobacteriota	58.6	57	1.8
Bdellovibrionota			
Deferribacterota			
Candidate OD1			
Candidate OP11			
Candidate OP3			
Deinococcota			
Candidate Latescibacterota	1.3		
Spirochaetota			
Mycoplasmatota (Tenericutes)			
Omnitrophicaeota			
Bipolaricaulota			
Patescibacterota (CPR group)		0.3	
Candidate Zixibacterota			
Candidate Methylomirabilota	1.5	0.8	2.7
Candidate Rokuibacteriota			
Verrucomicrobiota	2.2	1.3	2.7
Dadabacteriota			
Fusobacteriota			
Atribacterota			
Candidate TM6			
Elusimicrobiota			
DTB-120			
MBNT15			
WPS-2			
Archaea	2		1.1
Fungi			
UBP18			
Unclassified			
Other	2.5	0.6	4.5
Total	0	0	0
Depth			
Temperature			

Phylum	Shulgan-Tash Gray2Floor	Shulgan-Tash Olive	Shulgan-Tash WhiteRhizoidDense
Alphaproteobacteria	5	4	4.8
Betaproteobacteria			
Gammaproteobacteria	25.6	16.6	19
Campylobacterota			
Planctomycetota	8	5.4	3.6
Myxococcota	0.5		
Nitrospirota	1.1		
Gemmatimonadota	0.6	0.5	0.3
Armatimonadota	1	0.5	
Cyanobacteriota	0.2		
Chloroflexota	9.1	3	1.3
Firmicutes			
Chlorobiota			
Bacteroidota		0.5	
Acidobacteriota	11.2	8	4.6
Thermodesulfobacterota			
Actinobacteriota	24.4	51.6	60.4
Bdellovibrionota			
Deferribacterota			
Candidate OD1			
Candidate OP11			
Candidate OP3			
Deinococcota			
Candidate Latescibacterota	1	1.4	
Spirochaetota			
Mycoplasmatota (Tenericutes)			
Omnitrophicaeota			
Bipolaricaulota			
Patescibacterota (CPR group)		1.4	2.3
Candidate Zixibacterota			
Candidate Methylomirabilota	4.5	1.8	1.3
Candidate Rokuibacteriota			
Verrucomicrobiota	1.5	1.1	0.6
Dadabacteriota	0.2		
Fusobacteriota			
Atribacterota			
Candidate TM6			
Elusimicrobiota			
DTB-120			
MBNT15			
WPS-2			
Archaea	1.7	1.5	
Fungi			
UBP18			
Unclassified			
Other	4.4	2.7	1.8
Total	0	0	0
Depth			
Temperature			

Phylum	Shulgan-Tash WhiteRhizoidThin
Alphaproteobacteria	2.8
Betaproteobacteria	
Gammaproteobacteria	6
Campylobacterota	
Planctomycetota	3.2
Myxococcota	
Nitrospirota	
Gemmatimonadota	
Armatimonadota	
Cyanobacteriota	
Chloroflexota	2
Firmicutes	
Chlorobiota	
Bacteroidota	
Acidobacteriota	5
Thermodesulfobacterota	
Actinobacteriota	72.2
Bdellovibrionota	
Deferribacterota	
Candidate OD1	
Candidate OP11	
Candidate OP3	
Deinococcota	
Candidate Latescibacterota	1
Spirochaetota	
Mycoplasmatota (Tenericutes)	
Omnitrophicaeota	
Bipolaricaulota	
Patescibacterota (CPR group)	0.6
Candidate Zixibacterota	
Candidate Methylomirabilota	1
Candidate Rokuibacteriota	
Verrucomicrobiota	1.5
Dadabacteriota	
Fusobacteriota	
Atribacterota	
Candidate TM6	
Elusimicrobiota	
DTB-120	
MBNT15	
WPS-2	
Archaea	1.4
Fungi	
UBP18	
Unclassified	
Other	3.3
Total	0
Depth	
Temperature	

Supplementary Table 2. Information on how the abundances were calculated for each deep granitic or karst cave site with the links to the original works.

Name of the site	How the data were obtained	Manuscript	doi
**Deep granitic environments**			
BNO SSU	The classification and calculation of abundances based on raw Illumina reads with the RiboTaxa pipeline using the Silva v138 database release	this work	-
BNO	The GTDB-tk classification based on good-quality MAGs using the GTDB 207v2 release	this work	-
Boulby	The abundances were calculated on the basis of raw OTU counts from Supplementary Table 2	Bashir et al. Taxonomic and functional analyses of intact microbial communities thriving in extreme, astrobiology-relevant, anoxic sites. 2021, *Microbiome*	10.1186/s40168-020-00989-5
Nevada average	The abundances were calculated on the basis of raw OTU counts from Supplementary Table 5. Raw SSU counts were summed across all wells with sampling depths greater than 300 m (sampling depths were taken from Supplementary Table 2), and the final abundances were calculated on the basis of these summed counts	Merino et al. Subsurface microbial communities as a tool for characterizing regional-scale groundwater flow. 2022, *Science of the Total Environment*	10.1016/j.scitotenv.2022.156768
Chixulub	For granitic environments, the abundances were taken from the bar plot of Figure 4A. For the abundances of Alphaproteobacteria and Gammaproteobacteria, Figure 4B was used. The depth was averaged along 31 granitic sites from Supplementary Table 2	Quraish et al. Deep subsurface microbial life in impact-altered Late Paleozoic granitoid rocks from the Chicxulub impact crater. 2023, *Geobiology*	10.1111/gbi.12583
Deccan Traps	For granitic environments, the abundances were taken from the bar plots of Figure 2 (sites U7, U8, U9, P3, PV2, PV4). The depth was taken as an average between the six granitic sites mentioned	Dutta et al. Exploration of deep terrestrial subsurface microbiome in Late Cretaceous Deccan traps and underlying Archean basement, India. 2018, *Scientific Reports*	10.1038/s41598-018-35940-0
SURF D1	The abundances were taken from the bar plot of Supplementary Figure 1	Osburn et al. Contrasting Variable and Stable Subsurface Microbial Populations: an ecological time series analysis from the Deep Mine Microbial Observatory, South Dakota, USA. 2020, preprint	10.1101/2020.09.15.298141

SURF D2	The abundances were taken from the bar plot of Supplementary Figure 1	Osburn et al. Contrasting Variable and Stable Subsurface Microbial Populations: an ecological time series analysis from the Deep Mine Microbial Observatory, South Dakota, USA. 2020, preprint	10.1101/2020.09.15.298141
SURF D3	The abundances were taken from the bar plot of Supplementary Figure 1	Osburn et al. Contrasting Variable and Stable Subsurface Microbial Populations: an ecological time series analysis from the Deep Mine Microbial Observatory, South Dakota, USA. 2020, preprint	10.1101/2020.09.15.298141
SURF D4	The abundances were taken from the bar plot of Supplementary Figure 1	Osburn et al. Contrasting Variable and Stable Subsurface Microbial Populations: an ecological time series analysis from the Deep Mine Microbial Observatory, South Dakota, USA. 2020, preprint	10.1101/2020.09.15.298141
SURF D5	The abundances were taken from the bar plot of Supplementary Figure 1	Osburn et al. Contrasting Variable and Stable Subsurface Microbial Populations: an ecological time series analysis from the Deep Mine Microbial Observatory, South Dakota, USA. 2020, preprint	10.1101/2020.09.15.298141
SURF D6	The abundances were taken from the bar plot of Supplementary Figure 1	Osburn et al. Contrasting Variable and Stable Subsurface Microbial Populations: an ecological time series analysis from the Deep Mine Microbial Observatory, South Dakota, USA. 2020, preprint	10.1101/2020.09.15.298141
Aspo MM	The abundances were taken from Supplementary Table 6. The depth was calculated as an average across all MM sites	Lopez-Fernandez et al. Depth and Dissolved Organic Carbon Shape Microbial Communities in Surface Influenced but Not Ancient Saline Terrestrial Aquifers. 2018, *Frontiers in Mirobiology*	10.3389/fmicb.2018.02880
Aspo OS	The abundances were taken from Supplementary Table 6. The depth was calculated as an average across all OS sites	Lopez-Fernandez et al. Depth and Dissolved Organic Carbon Shape Microbial Communities in Surface Influenced but Not Ancient Saline Terrestrial Aquifers. 2018, *Frontiers in Mirobiology*	10.3389/fmicb.2018.02880

Aspo TM	The abundances were taken from Supplementary Table 6. The TM site was the only one with a depth of 448.4 m	Lopez-Fernandez et al. Depth and Dissolved Organic Carbon Shape Microbial Communities in Surface Influenced but Not Ancient Saline Terrestrial Aquifers. 2018, *Frontiers in Mirobiology*	10.3389/fmicb.2018.02880
Olkiluoto	The abundances were calculated using the data from Supplementary Table 3, column "Average"	Bell et al. Biogeochemical Cycling by a Low-Diversity Microbial Community in Deep Groundwater. 2018, *Frontiers in Microbiology*	10.3389/fmicb.2018.02129
Outokumpu 180m	The abundances were calculated using the data from Supplementary Table 2, "DNA" column. The number of archaeal sequences was 3 orders of magnitude smaller than that of the bacterial sequences (Table 2, main text, 16S rRNA OTUs counts). Thus, archaea shared the abundance of less than 0.1%, which is below the cutoff value of 0.2%, and so they were excluded from the final table of abundances	Purkamo et al. Microbial co-occurrence patterns in deep Precambrian bedrock fracture fluids. 2016, *Biogeosciences*	10.5194/bg-13-3091-2016
Outokumpu 500m	The abundances were calculated using the data from Supplementary Table 2, "DNA" column. The number of archaeal sequences was 3 orders of magnitude smaller than that of the bacterial sequences (Table 2, main text, 16S rRNA OTUs counts). Thus, archaea shared the abundance of less than 0.1%, which is below the cutoff value of 0.2%, and so they were excluded from the final table of abundances	Purkamo et al. Microbial co-occurrence patterns in deep Precambrian bedrock fracture fluids. 2016, *Biogeosciences*	10.5194/bg-13-3091-2016
Outokumpu 967m	The abundances were calculated using the data from Supplementary Table 2, "DNA" column. The number of archaeal sequences was 3 orders of magnitude smaller than that of the bacterial sequences (Table 2, main text, 16S rRNA OTUs counts). Thus, archaea shared the abundance of less than 0.1%, which is below the cutoff value of 0.2%, and so they were excluded from the final table of abundances	Purkamo et al. Microbial co-occurrence patterns in deep Precambrian bedrock fracture fluids. 2016, *Biogeosciences*	10.5194/bg-13-3091-2016
Outokumpu 1820m	The abundances were calculated using the data from Supplementary Table 2, "DNA" column. The number of archaeal sequences was 4 orders of magnitude smaller than that of the bacterial sequences (Table 2, main text, 16S rRNA OTUs counts). Thus, archaea shared the abundance of less than 0.01%, which is below the cutoff value of 0.2%, and so they were excluded from the final table of abundances	Purkamo et al. Microbial co-occurrence patterns in deep Precambrian bedrock fracture fluids. 2016, *Biogeosciences*	10.5194/bg-13-3091-2016

Outokumpu 2260m	The percentage of archaea and bacteria was calculated using the data from Table 2, main text, 16S rRNA OTUs counts. bacterial_abundance = 9.01*10^2/(9.01*10^2+2.32*10)*100=97.5%, archaeal_abundance = 2.32*10/(9.01*10^2+2.32*10)*100=2.5%. The abundances for bacterial phyla were calculated from Supplementary Table 2, "DNA" column and normalized by the value 0.975	Purkamo et al. Microbial co-occurrence patterns in deep Precambrian bedrock fracture fluids. 2016, *Biogeosciences*	10.5194/bg-13-3091-2016
Outokumpu 2300m	The abundances were calculated using the data from Supplementary Table 2, "DNA" column. The number of archaeal sequences was 3 orders of magnitude smaller than that of the bacterial sequences (Table 2, main text, 16S rRNA OTUs counts). Thus, archaea shared the abundance of less than 0.1%, which is below the cutoff value of 0.2%, and so they were excluded from the final table of abundances	Purkamo et al. Microbial co-occurrence patterns in deep Precambrian bedrock fracture fluids. 2016, *Biogeosciences*	10.5194/bg-13-3091-2016
Pyhäsalmi	From the raw OTU counts of Supplementary Table 2. The counts from all three repeats for DNA were summed up, and the phyla abundances were calculated. The same was done for RNA. The average of the DNA and RNA abundance values is the final abundance value	Purkamo et al. Ultradeep Microbial Communities at 4.4 km within Crystalline Bedrock: Implications for Habitability in a Planetary Context. 2020, *Life*	10.3390/life10010002
Otaniemi	From the raw OTU counts of Supplementary Table 2. The counts from all three repeats for DNA were summed up, and the phyla abundances were calculated	Purkamo et al. Ultradeep Microbial Communities at 4.4 km within Crystalline Bedrock: Implications for Habitability in a Planetary Context. 2020, *Life*	10.3390/life10010002
Grimsel	The abundances were calculated on the basis of the raw OTU counts from Supplementary Table 1. Only two columns "abBorehole_fluid_Y2_b" and "aaBorehole_fluid_Y2_a" were used	Engel et al. Stable microbial community in compacted bentonite after 5 years of exposure to natural granitic groundwater. 2023, *mSphere*	10.1128/msphere.00048-23
Kidd Creek FW12299 slide	The abundances obtained from Supplementary Tables 2 and 3	Wilpiszeski et al. In Situ Growth of Halophilic Bacteria in Saline Fracture Fluids from 2.4 km below Surface in the Deep Canadian Shield. 2020, *Life*	10.3390/life10120307
Kidd Creek FW12299 filter	The abundances obtained from Supplementary Tables 2 and 3	Wilpiszeski et al. In Situ Growth of Halophilic Bacteria in Saline Fracture Fluids from 2.4 km below Surface in the Deep Canadian Shield. 2020, *Life*	10.3390/life10120307
Kidd Creek FW12322 slide	The abundances obtained from Supplementary Tables 2 and 3	Wilpiszeski et al. In Situ Growth of Halophilic Bacteria in Saline Fracture Fluids from 2.4 km below Surface in the Deep Canadian Shield. 2020, *Life*	10.3390/life10120307

Kidd Creek FW12287 A filter	The abundances obtained from Supplementary Tables 2 and 3	Wilpiszeski et al. In Situ Growth of Halophilic Bacteria in Saline Fracture Fluids from 2.4 km below Surface in the Deep Canadian Shield. 2020, *Life*	10.3390/life10120307
Mizunami	The abundances were calculated on the basis of the raw OTU counts from Table S1.	Ino et al. Deep microbial life in high-quality granitic groundwater from geochemically and geographically distinct underground boreholes. 2016, *Environmental Microbiology Reports*	10.1111/1758-2229.12379
Dobrovo Inoye R-224	The abundances were taken from the bar plot of Figure 2 of the main text of the manuscript	Babich et al. Phylogenetic Diversity and Potential Activity of Bacteria and Fungi in the Deep Subsurface Horizons of an Uranium Deposit. 2021, *Microbiology*	10.1134/S0026261721040032
Dobrovo Inoye G-15	The abundances were taken from the bar plot of Figure 2 of the main text of the manuscript	Babich et al. Phylogenetic Diversity and Potential Activity of Bacteria and Fungi in the Deep Subsurface Horizons of an Uranium Deposit. 2021, *Microbiology*	10.1134/S0026261721040032
Severnyi D-1	The abundances were taken from Table 4 of the main text of the manuscript	Nazina et al. Microbiology of formation waters from the deep repository of liquid radioactive wastes Severnyi. 2004, *FEMS Microbiology Ecology*	10.1016/j.femsec.2004.02.017
Soudan	The abundances were taken from the Relative Abundance Table, available at https://doi.org/10.6073/pasta/6933c6fa8327eb7be3bd477d2ce978fd	Schuler et al. Densely populated biofilms and linked iron and sulfur cycles in the fractured-rock continental subsurface. 2024, *Geochimica et Cosmochimica Acta*	10.1016/j.gca.2024.04.019
Witwatersrand Be326	The abundances were taken from the bar plot of Figure 3 of the main text. The average for two years was calculated	Magnabosco et al. Comparisons of the composition and biogeographic distribution of the bacterial communities occupying South African thermal springs with those inhabiting deep subsurface fracture water. 2014, *Frontiers in Microbiology*	10.3389/fmicb.2014.00679
Witwatersrand Dr5IPC	The abundances were taken from the bar plot of Figure 3 of the main text of the manuscript	Magnabosco et al. Comparisons of the composition and biogeographic distribution of the bacterial	10.3389/fmicb.2014.00679

		communities occupying South African thermal springs with those inhabiting deep subsurface fracture water. 2014, *Frontiers in Microbiology*	
Witwatersrand FI88	The abundances were taken from the bar plot of Figure 3 of the main text of the manuscript	Magnabosco et al. Comparisons of the composition and biogeographic distribution of the bacterial communities occupying South African thermal springs with those inhabiting deep subsurface fracture water. 2014, *Frontiers in Microbiology*	10.3389/fmicb.2014.00679
Witwatersrand MM51940	The abundances were taken from the bar plot of Figure 3 of the main text of the manuscript	Magnabosco et al. Comparisons of the composition and biogeographic distribution of the bacterial communities occupying South African thermal springs with those inhabiting deep subsurface fracture water. 2014, *Frontiers in Microbiology*	10.3389/fmicb.2014.00679
Witwatersrand NO14	The abundances were taken from the bar plot of Figure 3 of the main text of the manuscript	Magnabosco et al. Comparisons of the composition and biogeographic distribution of the bacterial communities occupying South African thermal springs with those inhabiting deep subsurface fracture water. 2014, *Frontiers in Microbiology*	10.3389/fmicb.2014.00679
Witwatersrand TT109	The abundances were taken from the bar plot of Figure 3 of the main text of the manuscript	Magnabosco et al. Comparisons of the composition and biogeographic distribution of the bacterial communities occupying South African thermal springs with those inhabiting deep subsurface fracture water. 2014, *Frontiers in Microbiology*	10.3389/fmicb.2014.00679
**Cave environments**			
Borra caves	Abundances of phyla are given in text on pages 4 and 5 under the "Borra Caves Speleothems Microbial Community Structure" section.	Samanta, Sharma and Budhwar. Metagenome Analysis of Speleothem	10.1007/s00284-02

	Proteobacteria were divided into classes based on data from Figure 2A.	Microbiome from Subterranean Cave Reveals Insight into Community Structure, Metabolic Potential, and BGCs Diversity. 2023, *Current Microbiology*	3-03431-9
Yumugi	Abundances were manually extracted from bar plot of Figure 2B, main text of the manuscript, and Supllementary Figure S3C.	Turrini et al. The microbial community of a biofilm lining the wall of a pristine cave in Western New Guinea. 2020, *Microbiological Research*	10.1016/j.micres.2020.126584
Grotta grande C1	Abundances were calculated based on the data of Supplementary Table 2, then *Proteobacteria* were divided into classes using the classes ratio from Figure 4	Farda et al. Exploring structure, microbiota, and metagenome functions of epigean and hypogean black deposits by microscopic, molecular and bioinformatic approaches. 2022, *Scientific Reports*	10.1038/s41598-022-24159-9
Grotta grande C2	Abundances were calculated based on the data of Supplementary Table 2, then Proteobacteria were divided into classes using the classes ratio from Figure 5	Farda et al. Exploring structure, microbiota, and metagenome functions of epigean and hypogean black deposits by microscopic, molecular and bioinformatic approaches. 2022, *Scientific Reports*	10.1038/s41598-022-24159-9
Ovito di Pietrasecca C3	Abundances were calculated based on the data of Supplementary Table 2, then Proteobacteria were divided into classes using the classes ratio from Figure 6	Farda et al. Exploring structure, microbiota, and metagenome functions of epigean and hypogean black deposits by microscopic, molecular and bioinformatic approaches. 2022, *Scientific Reports*	10.1038/s41598-022-24159-9
Ovito di Pietrasecca C4	Abundances were calculated based on the data of Supplementary Table 2, then Proteobacteria were divided into classes using the classes ratio from Figure 7	Farda et al. Exploring structure, microbiota, and metagenome functions of epigean and hypogean black deposits by microscopic, molecular and bioinformatic approaches. 2022, *Scientific Reports*	10.1038/s41598-022-24159-9
Shulgan-Tash Azurite	Abundances were manually extracted from bar plot of Figure 4A, main text of the manuscript.	Gogoleva et al. Microbial tapestry of the Shulgan-Tash cave (Southern Ural, Russia): influences of environmental factors on the taxonomic composition of the cave biofilms. 2023, *Environmental Microbiome*	10.1186/s40793-023-00538-1
Shulgan-Tash	Abundances were manually extracted from bar plot of Figure 4A, main text of the manuscript.	Gogoleva et al. Microbial tapestry of the Shulgan-Tash	10.1186/s407

BrownWhite		cave (Southern Ural, Russia): influences of environmental factors on the taxonomic composition of the cave biofilms. 2023, *Environmental Microbiome*	93-023-00538-1
Shulgan-Tash Coralline	Abundances were manually extracted from bar plot of Figure 4A, main text of the manuscript.	Gogoleva et al. Microbial tapestry of the Shulgan-Tash cave (Southern Ural, Russia): influences of environmental factors on the taxonomic composition of the cave biofilms. 2023, *Environmental Microbiome*	10.1186/s40793-023-00538-2
Shulgan-Tash Gray1Floor	Abundances were manually extracted from bar plot of Figure 4A, main text of the manuscript.	Gogoleva et al. Microbial tapestry of the Shulgan-Tash cave (Southern Ural, Russia): influences of environmental factors on the taxonomic composition of the cave biofilms. 2023, *Environmental Microbiome*	10.1186/s40793-023-00538-3
Shulgan-Tash WhiteRhizoidFlat	Abundances were manually extracted from bar plot of Figure 4A, main text of the manuscript.	Gogoleva et al. Microbial tapestry of the Shulgan-Tash cave (Southern Ural, Russia): influences of environmental factors on the taxonomic composition of the cave biofilms. 2023, *Environmental Microbiome*	10.1186/s40793-023-00538-4
Shulgan-Tash WhiteWavyEdge	Abundances were manually extracted from bar plot of Figure 4A, main text of the manuscript.	Gogoleva et al. Microbial tapestry of the Shulgan-Tash cave (Southern Ural, Russia): influences of environmental factors on the taxonomic composition of the cave biofilms. 2023, *Environmental Microbiome*	10.1186/s40793-023-00538-5
Shulgan-Tash WhiteYellowEdge	Abundances were manually extracted from bar plot of Figure 4A, main text of the manuscript.	Gogoleva et al. Microbial tapestry of the Shulgan-Tash cave (Southern Ural, Russia): influences of environmental factors on the taxonomic composition of the cave biofilms. 2023, *Environmental Microbiome*	10.1186/s40793-023-00538-6
Shulgan-Tash CaveCu	Abundances were manually extracted from bar plot of Figure 4A, main text of the manuscript.	Gogoleva et al. Microbial tapestry of the Shulgan-Tash cave (Southern Ural, Russia):	10.1186/s40793-02

rd		influences of environmental factors on the taxonomic composition of the cave biofilms. 2023, *Environmental Microbiome*	3-005 38-7
Shulgan -Tash Gray2Fl oor	Abundances were manually extracted from bar plot of Figure 4A, main text of the manuscript.	Gogoleva et al. Microbial tapestry of the Shulgan-Tash cave (Southern Ural, Russia): influences of environmental factors on the taxonomic composition of the cave biofilms. 2023, *Environmental Microbiome*	10.118 6/s407 93-02 3-005 38-8
Shulgan -Tash Olive	Abundances were manually extracted from bar plot of Figure 4A, main text of the manuscript.	Gogoleva et al. Microbial tapestry of the Shulgan-Tash cave (Southern Ural, Russia): influences of environmental factors on the taxonomic composition of the cave biofilms. 2023, *Environmental Microbiome*	10.118 6/s407 93-02 3-005 38-9
Shulgan -Tash WhiteRh izoidDen se	Abundances were manually extracted from bar plot of Figure 4A, main text of the manuscript.	Gogoleva et al. Microbial tapestry of the Shulgan-Tash cave (Southern Ural, Russia): influences of environmental factors on the taxonomic composition of the cave biofilms. 2023, *Environmental Microbiome*	10.118 6/s407 93-02 3-005 38-10
Shulgan -Tash WhiteRh izoidThi n	Abundances were manually extracted from bar plot of Figure 4A, main text of the manuscript.	Gogoleva et al. Microbial tapestry of the Shulgan-Tash cave (Southern Ural, Russia): influences of environmental factors on the taxonomic composition of the cave biofilms. 2023, *Environmental Microbiome*	10.118 6/s407 93-02 3-005 38-11

Supplementary Table 3. Distribution of genes in MAGs.

BIN №	Aromatics degradation	Complex carbon degradation						
	ubiX	Chitinase	Hexosaminidase	beta-glucosidase	cellulase	arabinosidase	alpha-amylase	glucoamylase
bin.1								
bin.5								
bin.6								
bin.14	+		+		+	+	+	
bin.15								
bin.16								
bin.17			+				+	
bin.18								
bin.19								
bin.20	+		+					
bin.22							+	+
bin.24		+	+					
bin.25								
bin.29								
bin.30	+							
bin.37								
bin.40								
bin.41								
bin.42								

BIN №		Oxydative phosphorylation						
	isoamylase	coxA	coxB	ccoN	ccoO	ccoP	nuoA	nuoB
bin.1		+	+				+	+
bin.5					+		+	+
bin.6		+	+	+	+	+	+	+
bin.14	+			+	+		+	+
bin.15		+	+			+	+	+
bin.16				+	+		+	+
bin.17				+	+		+	+
bin.18				+	+	+	+	+
bin.19		+	+	+	+	+	+	+
bin.20		+	+				+	+
bin.22	+	+		+	+	+	+	+
bin.24		+					+	+
bin.25				+	+	+	+	+
bin.29		+				+	+	+
bin.30		+	+	+	+	+	+	+
bin.37		+	+	+	+	+	+	+
bin.40				+	+	+	+	+
bin.41		+	+				+	+
bin.42		+	+	+	+	+	+	+

BIN №								
	nuoC	sdhC	sdhD	petA	petB	atpA	atpD	cydA
bin.1	+	+	+	+	+	+	+	
bin.5	+	+	+		+	+	+	+
bin.6	+	+	+	+	+	+	+	
bin.14						+	+	
bin.15		+	+	+	+	+	+	+
bin.16					+		+	+
bin.17	+	+				+	+	
bin.18						+	+	
bin.19	+	+	+	+	+	+	+	+
bin.20					+	+	+	
bin.22		+				+	+	
bin.24	+	+	+	+	+	+	+	+
bin.25	+				+	+	+	+
bin.29		+			+	+	+	
bin.30		+				+	+	
bin.37	+	+	+	+	+	+	+	
bin.40	+	+	+		+	+	+	+
bin.41	+	+	+	+	+	+	+	
bin.42		+				+	+	

BIN №		Entner-Doudoroff	Methylotrophy					
	cydB	KDPG aldolase	mxaF	mauA	mauB	fae	frmA	fdoG
bin.1								
bin.5								+
bin.6							+	+
bin.14								
bin.15	+					+		+
bin.16								
bin.17								
bin.18			+	+	+	+	+	
bin.19	+							
bin.20								
bin.22								
bin.24	+	+						
bin.25	+							
bin.29								
bin.30								
bin.37			+	+	+		+	+
bin.40								
bin.41								
bin.42			+					

BIN №								
	fdoH	fdwB	coxS	coxM	coxL	pmoA	pmoB	pmoC
bin.1								
bin.5								
bin.6	+	+	+	+	+			
bin.14								
bin.15		+						+
bin.16								
bin.17								
bin.18								
bin.19								
bin.20								
bin.22								
bin.24								
bin.25								
bin.29			+	+				
bin.30								
bin.37		+						
bin.40								
bin.41								
bin.42								

BIN №			Carbon fixation					
	mmoB	mmoD	RuBisCo form I	RuBisCo form II	cdhD	cdhE	cooS	aclA
bin.1			+					
bin.5					+	+	+	
bin.6								
bin.14								
bin.15			+					
bin.16								+
bin.17								
bin.18								
bin.19								
bin.20								
bin.22								
bin.24								
bin.25								+
bin.29								
bin.30								
bin.37	+							
bin.40					+	+	+	
bin.41			+					
bin.42								

BIN №		Hydrogenases				Nitrogen cycling		
	aclB	NiFe-group1	NiFe-group2	NiFe-group3	NiFe-group4	UreA	UreB	UreC
bin.1				+		+	+	+
bin.5				+				
bin.6								
bin.14								
bin.15		+	+					
bin.16	+							
bin.17								
bin.18		+						
bin.19		+						
bin.20								
bin.22								
bin.24								
bin.25	+		+		+			
bin.29								
bin.30								
bin.37								
bin.40								
bin.41				+		+	+	+
bin.42		+			+			

BIN №								
	nxrA	nxrB	NapA	NapB	nrfH	nrfA	NosZ	NifD
bin.1								
bin.5		+			+	+		
bin.6								
bin.14								
bin.15								+
bin.16	+				+	+		
bin.17	+	+	+	+	+	+		
bin.18						+	+	
bin.19								
bin.20								
bin.22							+	
bin.24								
bin.25	+							
bin.29						+	+	
bin.30			+	+			+	
bin.37								
bin.40	+	+			+			
bin.41								
bin.42			+	+			+	

BIN №								
	NifK	NifH	amoA	amoB	amoC	narG	narH	nirS
bin.1					+			
bin.5								
bin.6								
bin.14								
bin.15	+	+						
bin.16								
bin.17								
bin.18								
bin.19								
bin.20								
bin.22								
bin.24								
bin.25	+							
bin.29		+				+	+	+
bin.30								
bin.37						+	+	+
bin.40								
bin.41					+			
bin.42						+	+	

BIN №						Iron Cycling		
	nirD	nirK	norB	norC	hzoA	FoxE	FoxZ	MtoA
bin.1		+	+	+				
bin.5				+	+	+		
bin.6								
bin.14								
bin.15								
bin.16	+	+						
bin.17				+				
bin.18			+	+				+
bin.19								
bin.20								
bin.22								
bin.24								
bin.25				+				
bin.29								
bin.30								
bin.37			+				+	+
bin.40				+	+	+		
bin.41		+	+	+				
bin.42								

BIN №								
	MtrA	MtrB	MtrC	Cyc1	Cyc2	DmkA	DmkB	FmnB
bin.1				+			+	+
bin.5							+	+
bin.6								
bin.14							+	
bin.15								
bin.16							+	
bin.17							+	+
bin.18	+	+					+	+
bin.19	+	+	+				+	+
bin.20							+	+
bin.22							+	
bin.24								
bin.25							+	
bin.29						+	+	+
bin.30							+	+
bin.37	+	+						
bin.40							+	+
bin.41				+			+	+
bin.42					+		+	

BIN №								
	Ndh2	DFE_0448	DFE_0449	DFE_0450	DFE_0451	DFE_0461	DFE_0462	DFE_0463
bin.1								
bin.5		+	+		+			
bin.6								
bin.14	+							
bin.15								
bin.16	+	+			+			+
bin.17	+	+	+		+	+	+	+
bin.18	+	+	+		+	+	+	
bin.19		+	+			+	+	
bin.20	+				+			
bin.22					+			
bin.24								
bin.25	+				+			+
bin.29	+							
bin.30								
bin.37		+	+			+	+	
bin.40								
bin.41								
bin.42	+	+	+					

BIN №		Halogenated compound utilization	Arsenate cycling				Sulfur cycling
	DFE_0465	2-haloacid dehalogenase	arrA	arsC	arsM	aioA	sdo
bin.1							
bin.5							
bin.6		+	+	+		+	+
bin.14							+
bin.15							+
bin.16							
bin.17							
bin.18		+		+			+
bin.19	+						
bin.20							
bin.22		+					
bin.24							
bin.25							+
bin.29	+						+
bin.30		+					
bin.37							
bin.40							
bin.41							
bin.42	+		+		+		

BIN №								
	soxC	soxD	soxX	soxYZ	soxZ	sqr	dsrH	aprA
bin.1								
bin.5								
bin.6								+
bin.14								
bin.15	+	+		+	+	+	+	
bin.16								
bin.17								
bin.18								
bin.19								
bin.20								
bin.22								
bin.24								
bin.25	+	+	+			+		
bin.29								
bin.30								
bin.37								
bin.40								
bin.41								
bin.42								

BIN №				Perchlorate reduction	
	aprB	phsA	sat	pcrA	pcrB
bin.1					
bin.5					
bin.6	+	+	+		
bin.14					
bin.15					
bin.16					
bin.17					
bin.18					
bin.19					
bin.20					
bin.22					
bin.24					
bin.25					
bin.29				+	+
bin.30					
bin.37					
bin.40					
bin.41					
bin.42					

Supplementary Figure 1. Geological map of the Andyrchy mountain, adopted from (Sobissevitch AL, Gridnev DG, Sobissevitch LE, Kanonidi KK. 2008. Instrumental equipment of geophysical observatory at North Caucasus. Seism Instruments https://doi.org/10.3103/s0747923908010027), with the sampling site (red circle) in the farthermost part of the tunnel, 4.2 km from the entrance (black rectangle).

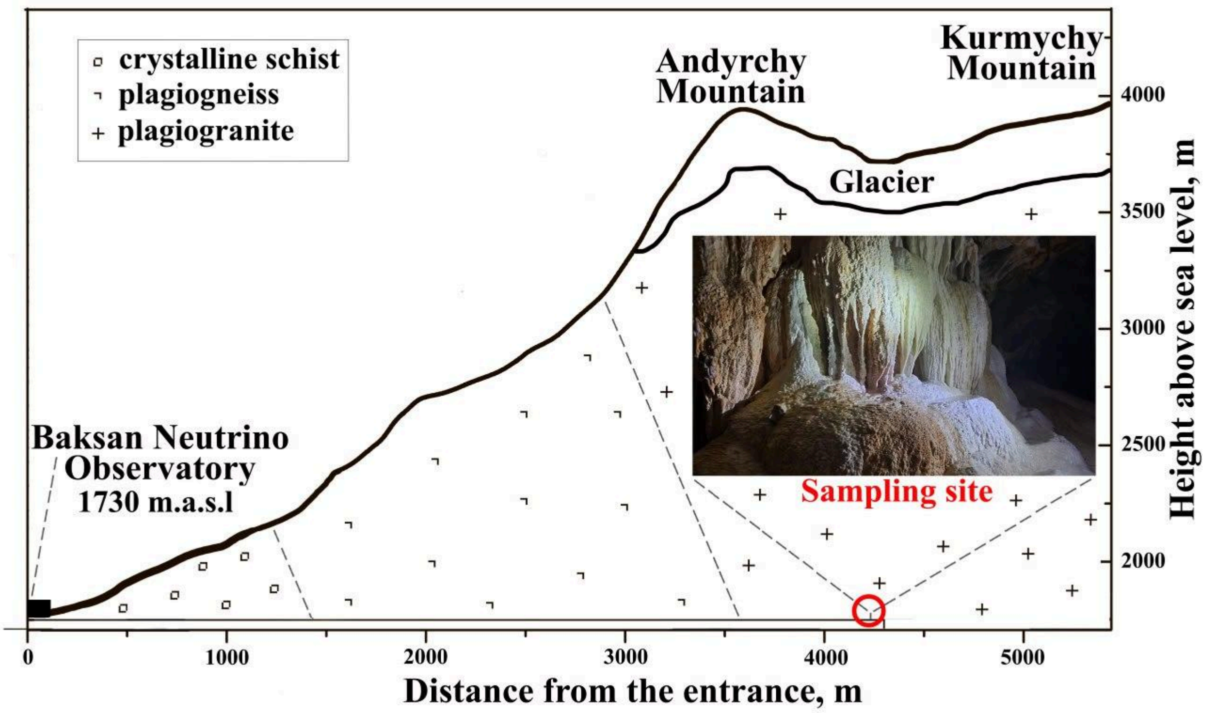

Supplementary Figure 2. Map of deep granitic and karst cave sites with microbial communities used in this study.

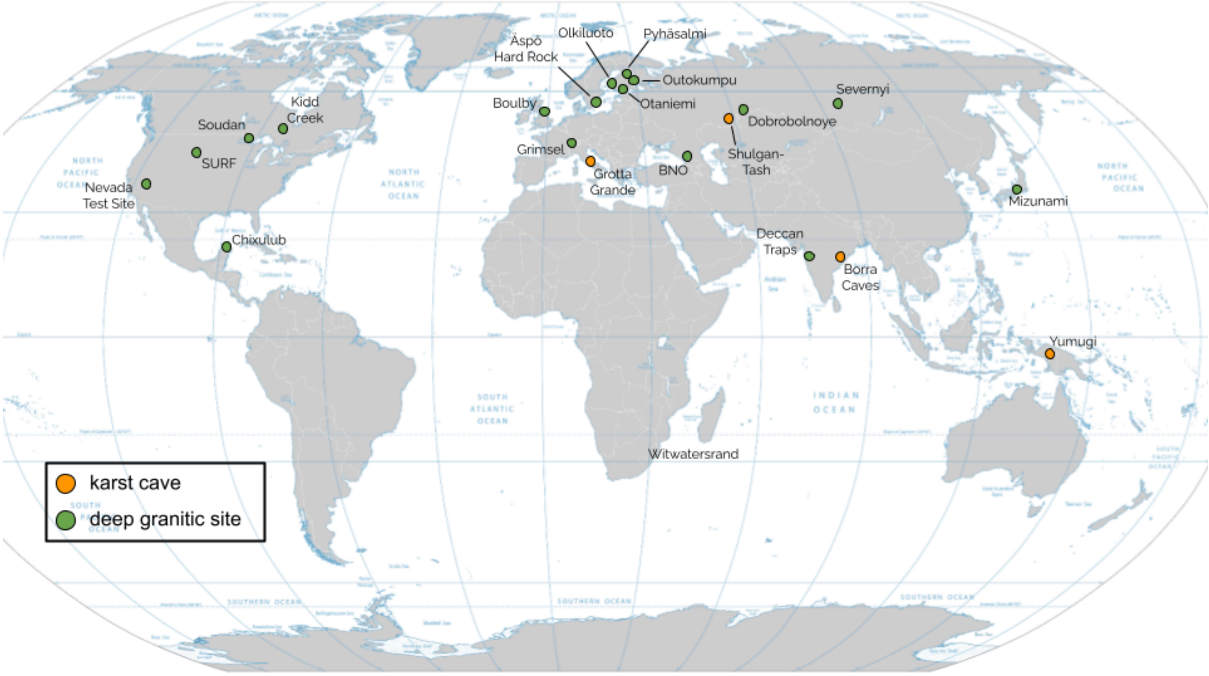

Supplementary Figure 3. Distribution of secondary metabolite biosynthetic gene clusters by phylum.

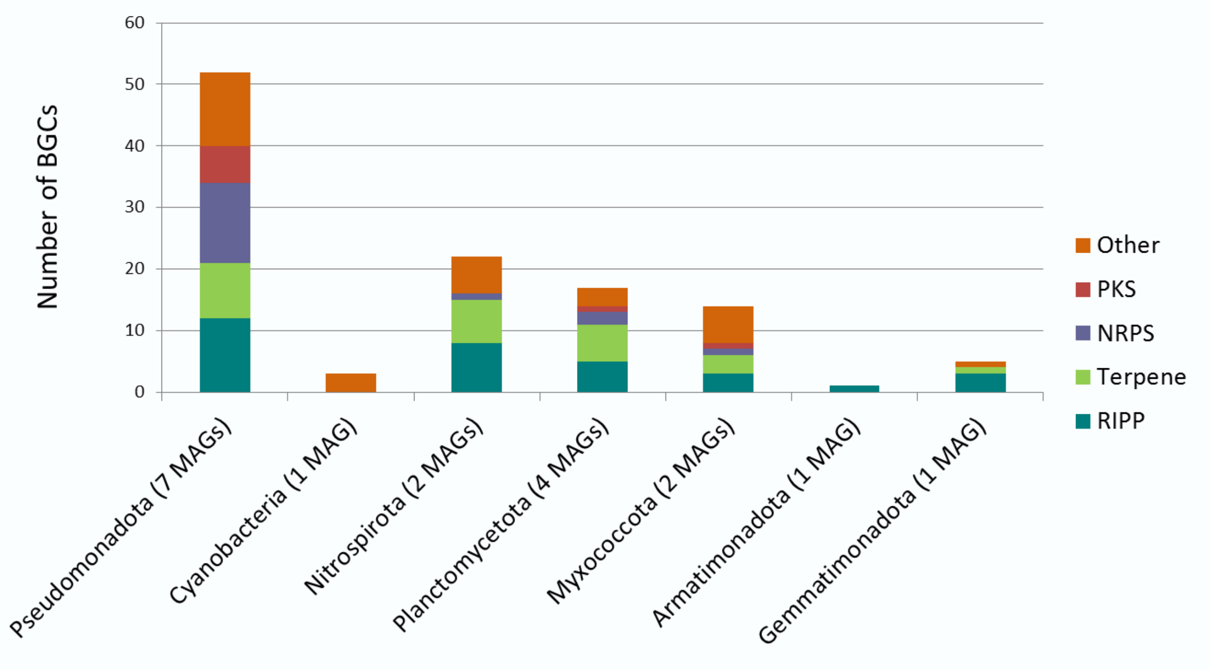

Supplementary Figure 4. Phylogeny of putative genera. For each MAG that was putatively marked by GTDB-Tk as a new genus on the trees produced by GTDB-Tk closest relatives that came from RefSeq database were located. ANI values between that node and MAG putatively of the new genus were added on the tree nodes. We propose six new genera of bacteria, namely "*Candidatus* Jinrbaksania", "*Candidatus* Neutrinellum", "*Candidatus* Jinrextremum", "*Candidatus* Inrsubterrania", "*Candidatus* Inralta", "*Candidatus* Neutrinobacter".

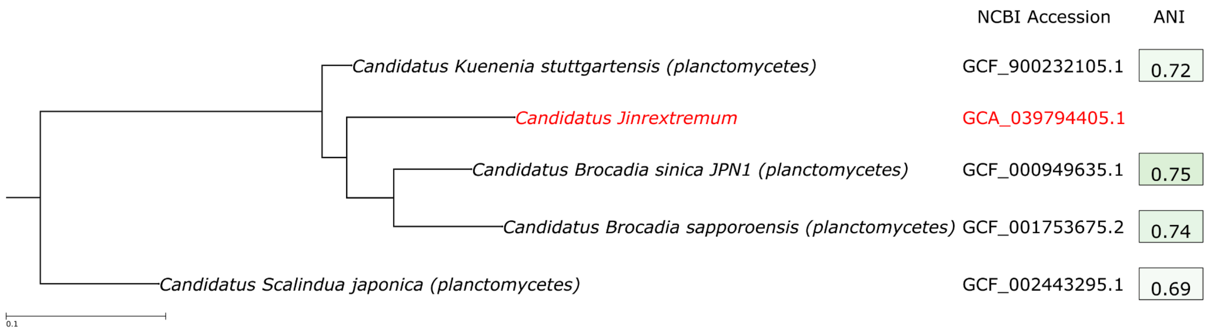

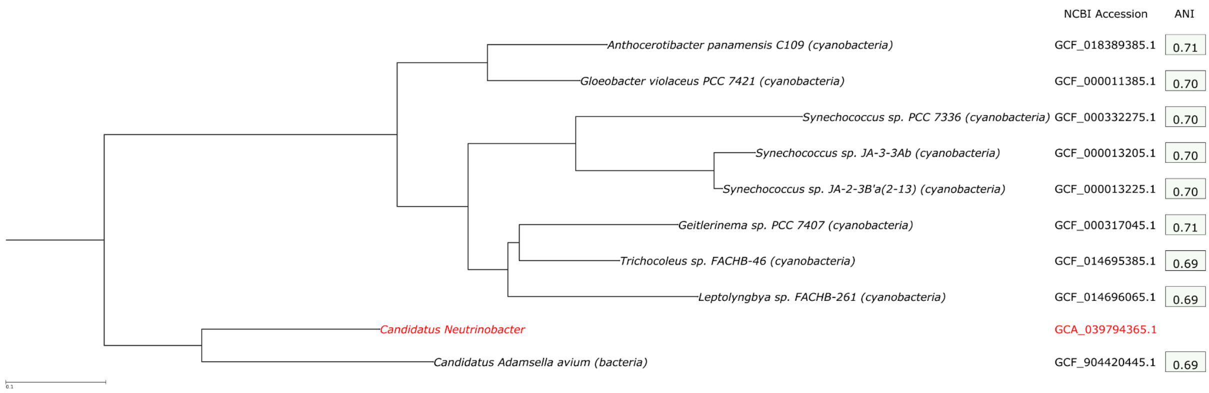

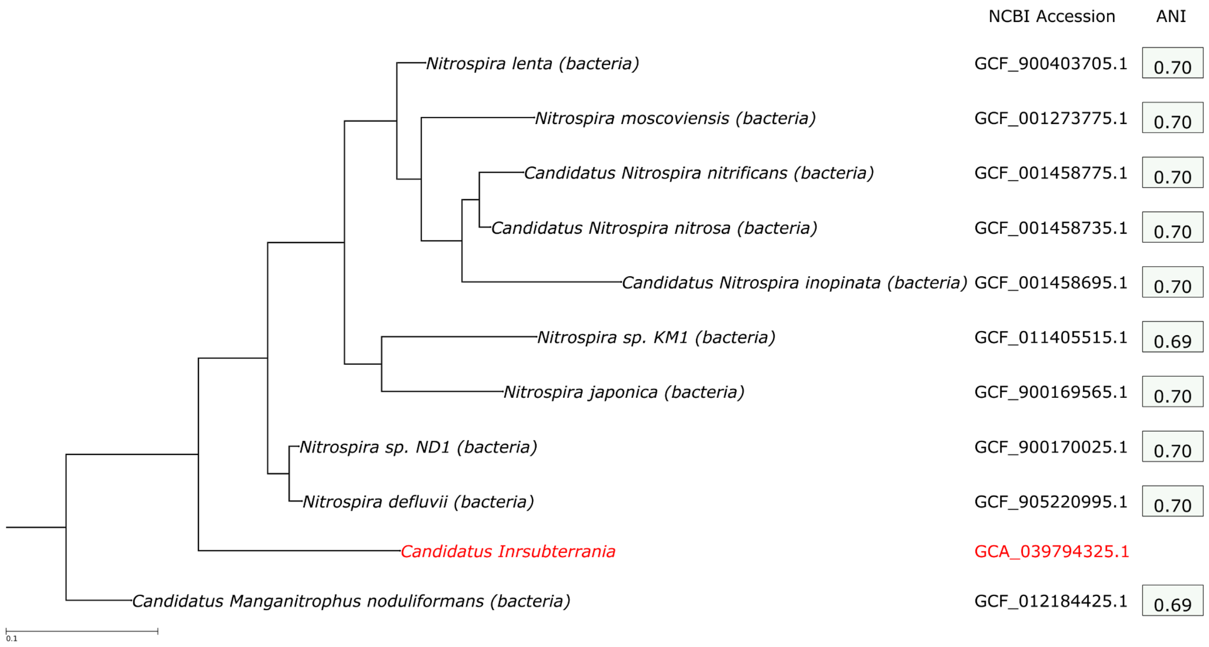
NCBI Accession
ANI
Nitrospira lenta (bacteria) GCF_900403705.1 0.70
Nitrospira moscoviensis (bacteria) GCF_001273775.1 0.70
Candidatus Nitrospira nitrificans (bacteria) GCF_001458775.1 0.70
Candidatus Nitrospira nitrosa (bacteria) GCF_001458735.1 0.70
Candidatus Nitrospira inopinata (bacteria) GCF_001458695.1 0.70
Nitrospira sp. KM1 (bacteria) GCF_011405515.1 0.69
Nitrospira japonica (bacteria) GCF_900169565.1 0.70
Nitrospira sp. ND1 (bacteria) GCF_900170025.1 0.70
Nitrospira defluvii (bacteria) GCF_905220995.1 0.70
Candidatus Inrsubterrania GCA_039794325.1
Candidatus Manganitrophus noduliformans (bacteria) GCF_012184425.1 0.69
0.1

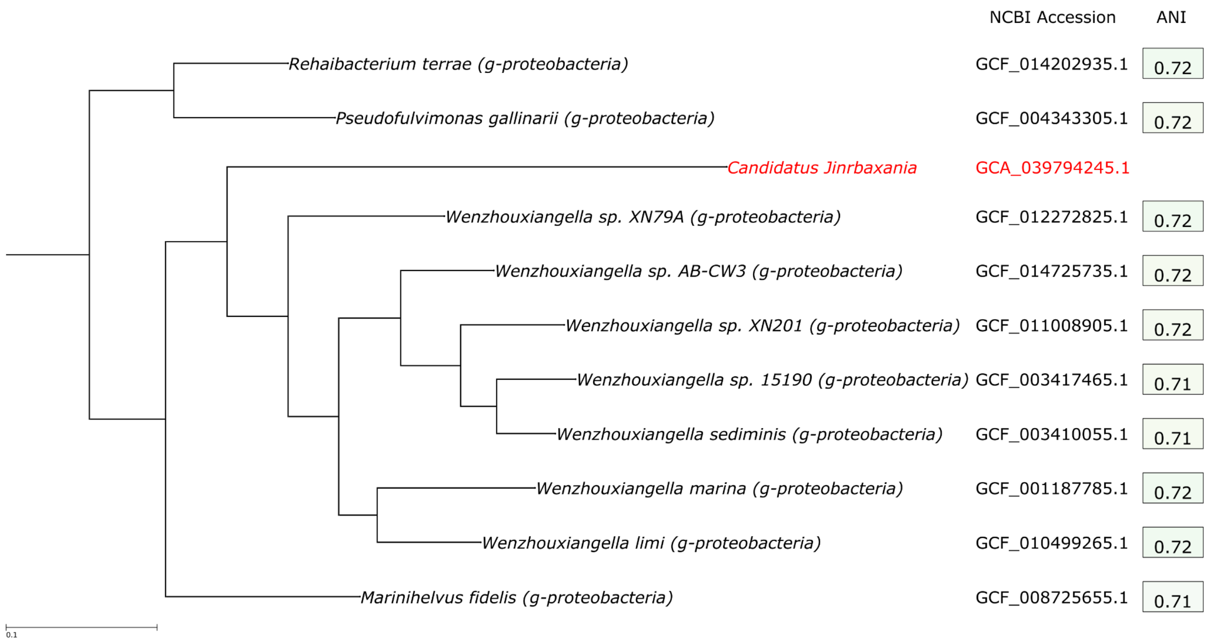
NCBI Accession
ANI
Rehaibacterium terrae (g-proteobacteria) GCF_014202935.1 0.72
Pseudofulvimonas gallinarii (g-proteobacteria) GCF_004343305.1 0.72
Candidatus Jinrbaxania GCA_039794245.1
Wenzhouxiangella sp. XN79A (g-proteobacteria) GCF_012272825.1 0.72
Wenzhouxiangella sp. AB-CW3 (g-proteobacteria) GCF_014725735.1 0.72
Wenzhouxiangella sp. XN201 (g-proteobacteria) GCF_011008905.1 0.72
Wenzhouxiangella sp. 15190 (g-proteobacteria) GCF_003417465.1 0.71
Wenzhouxiangella sediminis (g-proteobacteria) GCF_003410055.1 0.71
Wenzhouxiangella marina (g-proteobacteria) GCF_001187785.1 0.72
Wenzhouxiangella limi (g-proteobacteria) GCF_010499265.1 0.72
Marinihelvus fidelis (g-proteobacteria) GCF_008725655.1 0.71
0.1

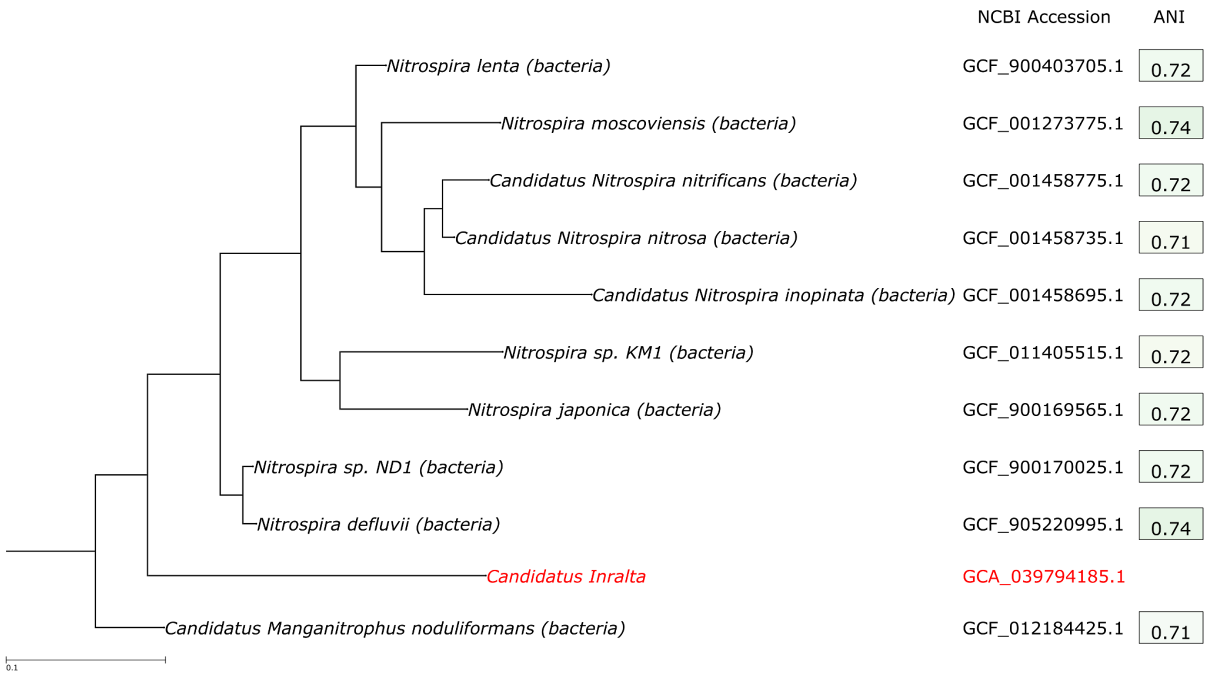

NCBI Accession
ANI
Nitrospira lenta (bacteria)
GCF_900403705.1
0.72
Nitrospira moscoviensis (bacteria)
GCF_001273775.1
0.74
Candidatus Nitrospira nitrificans (bacteria)
GCF_001458775.1
0.72
Candidatus Nitrospira nitrosa (bacteria)
GCF_001458735.1
0.71
Candidatus Nitrospira inopinata (bacteria)
GCF_001458695.1
0.72
Nitrospira sp. KM1 (bacteria)
GCF_011405515.1
0.72
Nitrospira japonica (bacteria)
GCF_900169565.1
0.72
Nitrospira sp. ND1 (bacteria)
GCF_900170025.1
0.72
Nitrospira defluvii (bacteria)
GCF_905220995.1
0.74
Candidatus Inralta
GCA_039794185.1
Candidatus Manganitrophus noduliformans (bacteria)
GCF_012184425.1
0.71
0.1

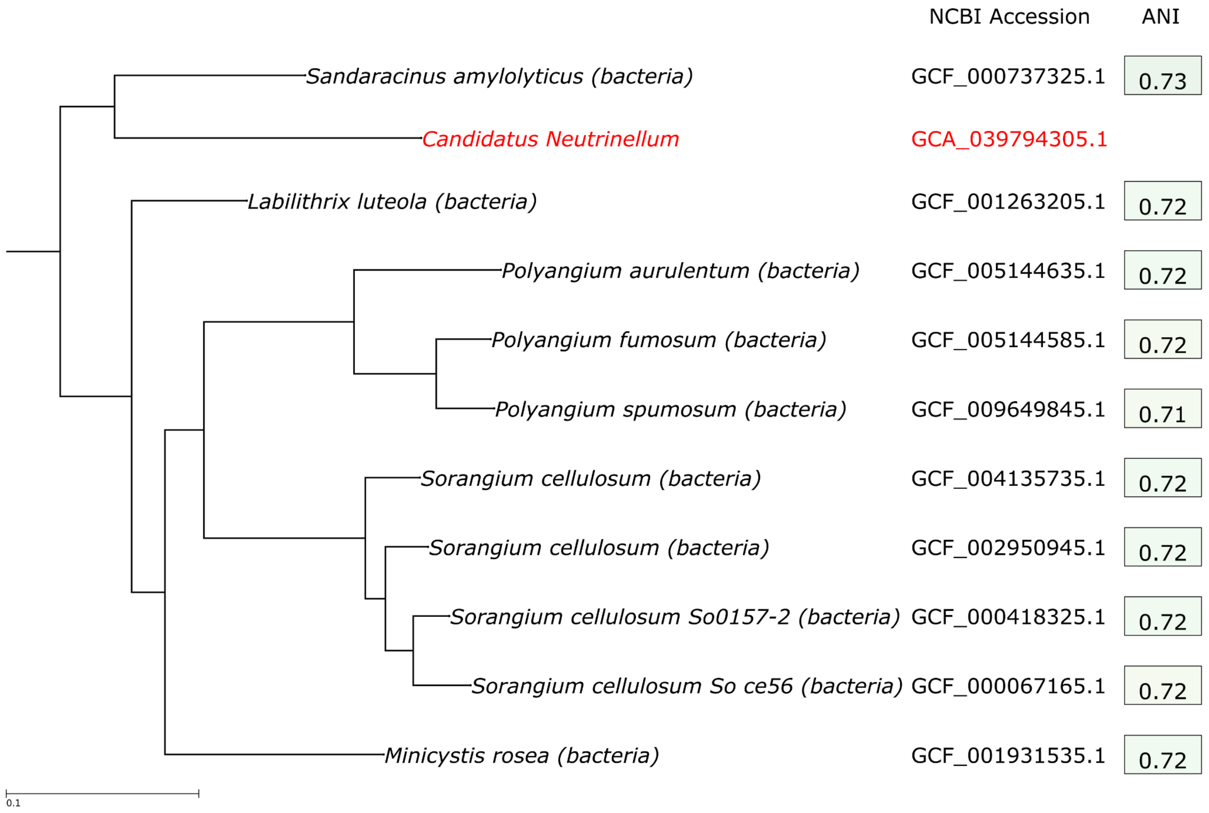

NCBI Accession
ANI
Sandaracinus amylolyticus (bacteria)
GCF_000737325.1
0.73
Candidatus Neutrinellum
GCA_039794305.1
Labilithrix luteola (bacteria)
GCF_001263205.1
0.72
Polyangium aurulentum (bacteria)
GCF_005144635.1
0.72
Polyangium fumosum (bacteria)
GCF_005144585.1
0.72
Polyangium spumosum (bacteria)
GCF_009649845.1
0.71
Sorangium cellulosum (bacteria)
GCF_004135735.1
0.72
Sorangium cellulosum (bacteria)
GCF_002950945.1
0.72
Sorangium cellulosum So0157-2 (bacteria)
GCF_000418325.1
0.72
Sorangium cellulosum So ce56 (bacteria)
GCF_000067165.1
0.72
Minicystis rosea (bacteria)
GCF_001931535.1
0.72
0.1
